# Hepatitis B virus X protein (HBx) enhances centrosomal P4.1-associated protein (CPAP) expression to promote hepatocarcinogenesis

**DOI:** 10.1186/s12929-019-0534-9

**Published:** 2019-06-06

**Authors:** Chia-Jui Yen, Shu-Ting Yang, Ruo-Yu Chen, Wenya Huang, Kazuaki Chayama, Ming-Hao Lee, Shiang-Jie Yang, Hong-Sheng Lai, Hsin-Yi Yen, Yu-Wei Hsiao, Ju-Ming Wang, Yih-Jyh Lin, Liang-Yi Hung

**Affiliations:** 1 Department of Biotechnology and Bioindustry Sciences, College of Bioscience and Biotechnology, National Cheng Kung University, Tainan, 70101 Taiwan; 2 Institute of Bioinformatics and Biosignal Transduction, College of Bioscience and Biotechnology, National Cheng Kung University, Tainan, 70101 Taiwan; 3 Department of Pharmacology, National Cheng Kung University, Tainan, 70101 Taiwan; 4 Institute of Basic Medical Sciences, National Cheng Kung University, Tainan, 70101 Taiwan; 5 Department of Medical Laboratory Science and Biotechnology, College of Medicine, National Cheng Kung University, Tainan, 70101 Taiwan; 6 Division of Hematology and Oncology, Department of Internal Medicine, National Cheng Kung University Hospital, College of Medicine, National Cheng Kung University, Tainan, 70101 Taiwan; 7 Division of General and Transplantation Surgery, Department of Surgery, National Cheng Kung University Hospital, College of Medicine, National Cheng Kung University, Tainan, 70101 Taiwan; 8 Ph.D. Program for Cancer Molecular Biology and Drug Discovery, College of Medical Science and Technology, Taipei Medical University, Taipei, 11031 Taiwan; 9 Graduate Institute of Medicine, College of Medicine, Kaohsiung Medical University, Kaohsiung, 80708 Taiwan; 10 Department of Gastroenterology and Metabolism, Applied Life Sciences, Institute of Biomedical & Health Sciences, Hiroshima University, Hiroshima, 734-8551 Japan

**Keywords:** Hepatocarcinogenesis, CPAP, HBx, NF-κB, Inflammation

## Abstract

**Background:**

Our previous report suggested that centrosomal P4.1-associated protein (CPAP) is required for Hepatitis B virus (HBV) encoded non-structure protein X (HBx)-mediated nuclear factor kappa light chain enhancer of activated B cells (NF-κB) activation. CPAP is overexpressed in HBV-associated hepatocellular carcinoma (HCC); however, the interaction between CPAP and HBx in HBV-HCC remains unclear.

**Methods:**

The mRNA expression of *CPAP* and *HBx* was analyzed by quantitative-PCR (Q-PCR). NF-κB transcriptional activity and *CPAP* promoter activity were determined using a reporter assay in Huh7 and Hep3B cells. Immunoprecipitation (IP) and in situ proximal ligation assay (PLA) were performed to detect the interaction between CPAP and HBx. Chromatin-IP was used to detect the association of cAMP response element binding protein (CREB) and HBx with the *CPAP* promoter. Cell proliferation was measured using cell counting kit CCK-8, Bromodeoxyuridine (5-bromo-2′-deoxyuridine, BrdU) incorporation, and clonogenic assays. The tumorigenic effects of CPAP were determined using xenograft animal models.

**Results:**

HBx can transcriptionally up-regulate *CPAP* via interacting with CREB. Overexpressed CPAP directly interacted with HBx to promote HBx-mediated cell proliferation and migration; SUMO modification of CPAP was involved in interacting with HBx. Knocked-down expression of CPAP decreased the HBx-mediated tumorigenic effects, including cytokines secretion. Interestingly, overexpressed CPAP maintained the HBx protein stability in an NF-κB-dependent manner; and the expression levels of CPAP and HBx were positively correlated with the activation status of NF-κB in HCC. Increased expression of *CPAP* and *CREB* mRNAs existed in the high-risk group with a lower survival rate in HBV-HCC.

**Conclusion:**

The interaction between CPAP and HBx can provide a microenvironment to facilitate HCC development via enhancing NF-κB activation, inflammatory cytokine production, and cancer malignancies. This study not only sheds light on the role of CPAP in HBV-associated HCC, but also provides CPAP as a potential target for blocking the hyper-activated NF-κB in HCC.

**Electronic supplementary material:**

The online version of this article (10.1186/s12929-019-0534-9) contains supplementary material, which is available to authorized users.

## Background

Hepatitis B virus (HBV) infection is a major etiological factor in acute and chronic hepatitis, and enhances the development of liver diseases such as cirrhosis and hepatocellular carcinoma (HCC). Among the different HBV proteins encoded by HBV genome, the X protein of HBV (HBx) plays a critical role in HBV-associated HCC development possibly through triggering specific oncogenic pathways and causing an accumulation of genetic and epigenetic alterations in regulatory genes [3, 15, 20, 26].

HBx is a multifunctional protein that modulates the expression of various cellular and viral genes involving in cell survival, cell cycle progression, DNA repair, invasion, protein degradation, and regulates several signaling pathways such as Ras/Raf/MAPK, PI3K/Akt, NF-κB, and JNK [9, 12, 17, 25, 36, 38]. Additionally, functions of HBx on cytoplasmic signal transduction cascades and transcriptional activation imply that HBx is both a cytoplasmic and nuclear protein [18]. It is known that HBx serves as a powerful transcriptional activator that up-regulates several transcription factors including nuclear factor kappa light chain enhancer of activated B cells (NF-κB), activator protein 1 (AP-1), and even the HBV genome [21, 31]. However, HBx does not bind DNA directly. HBx affects transcription by interacting with several transcription factors, including DNA-binding factors and complexes of transcriptional machinery [2]. For example, HBx interacts with TATA box-binding protein (TBP), RNA polymerase II subunit 5 (RPB5), and transcription factor IIB (TFIIB) to regulate RNA polymerase transcription [4, 16, 32]. In the nucleus, HBx associates with C-terminal binding protein (CBP/p300) and binds to the cAMP response element binding protein (CREB)-targeting site of the promoters of *interleukin-8 (IL-8)* and proliferating cell nuclear antigen (*PCNA*) [7, 35]. Recently, an *HBx* transgenic mouse model showed a high incidence of liver tumor formation without fibrosis in 90% of cases and has been widely used as an animal model for studying the detailed mechanisms of chronic HBV infection in HCC development [24, 30]. Although the role of HBx in the pathogenesis of HCC is well understood, the mechanism by which HBx regulates the gene expression network is not fully clear.

Previously, we showed that the expression of centrosomal P4.1-associated protein (CPAP) in HBV-associated HCC correlates with a poor prognosis [34]. CPAP has been reported to be part of the γ-tubulin complex, which is associated with γ-tubulin in both the centrosomal and cytosolic fractions throughout the cell cycle, and plays an essential role in microtubule nucleation and procentriole elongation [6, 10, 28]. Interestingly, CPAP also regulates cell apoptosis and the growth of neural precursor cells [8, 29]. There are three nuclear localization signals and two nuclear export signals within the CPAP polypeptide [23], indicating CPAP can shuttle between the nucleus and cytoplasm. Furthermore, CPAP has been shown to act as a transcriptional coactivator of signal transducer and activator of transcription 5 (STAT5) and NF-κB [13, 23]. TNF-α-induced small ubiquitin-like modifier (SUMO) modification of CPAP is required for IκB kinase (IKK)-mediated NF-κB activation in HCC cell lines and promotes the growth of HCC cells, suggesting that CPAP is critical for the association between NF-κB and inflammation-related diseases, such as HCC [34]. In addition, the cooperation of CPAP and HBx in regulating the transcriptional activity of NF-κB, provides evidence that CPAP plays an important role in HBx-mediated HCC development [34]. However, the relationship between CPAP and HBx, and the physiological roles of CPAP in HBV-associated HCC are still unclear.

In this study, we investigated the interaction between CPAP and HBx and determined the functional roles of the CPAP-HBx interaction in HBx-mediated hepatocarcinogenesis. Our results indicated that HBx transcriptionally increased the expression of *CPAP* via interacting with CREB, and overexpressed CPAP increased the protein stability of HBx in an NF-κB-dependent manner, both of which resulted in an increased activity and target gene expression of NF-κB. The reciprocal regulatory loop between CPAP and HBx at the transcriptional and post-transcriptional levels presents a complex relationship during early and late hepatocarcinogenesis, as well as contributes an HCC-promoting microenvironment, in HBV-related HCC. HCC patients with co-overexpressed *CREB* and *CPAP* mRNAs have a poor prognostic value. Taken together, our results provide strong evidence that CPAP is crucial for HBx-induced HCC development, therefore offering opportunities to develop mechanism-based therapies.

## Methods

### Cell culture, reagents, and antibodies

HCC cell lines Huh7, SK-Hep1, Hep3B, Hep3BX, HepG2, and HepG2X were maintained in Dulbecco’s modified Eagle’s medium (DMEM, Sigma, St. Louis, MO). Hep3BX and HepG2X are HBx stably expressed Hep3B and HepG2 cells, respectively. HepAD38/Tet-off cells [14] were grown in DMEM supplemented with 200 mM GlutaMax (Gibco, Carlsbad, CA), 0.3 μg/ml doxycycline, and 400 μg/ml G418. HepG2-hNTCP-C4 cells [5] were cultured with DMEM/F-12 (Sigma) with 200 mM GlutaMax, 50 μM hydrocortisone, 5 μg/ml insulin, and 400 μg/ml G418. Media was supplemented with 10% fetal bovine serum, 100 μg/ml streptomycin, and 100 units/ml penicillin. The cells were incubated at 37 °C in a humidified chamber containing 5% CO_2_. Human TNF-α, cycloheximide, and anti-α-tubulin antibodies were purchased from Sigma. The antibody against GFP was purchased from Clontech (Palo Alto, CA). Antibody against HA was purchased from Covance (Berkeley, CA). Antibodies against GAPDH and NF-κB/p65 were purchased from Santa Cruz Biotechnology (Santa Cruz, CA). The ON-TARGET plus SMARTpool siRNA for knockdown of CPAP and NF-κB/p65 were obtained from Dharmacon RNA Technologies (Lafayette, CO). sh*GFP* and sh*CREB* were obtained from National RNAi core facility in Taiwan.

### Xenograft tumorigenicity assay

Hep3B cells (2 × 10^6^) stably expressed GFP, GFP-CPAP/WT, GFP-CPAP/MT [34] were injected subcutaneously into the right flank region of 5-week-old male NOD-SCID mice. The tumor volumes were measured using calipers every 3 days. Tumor size was measured using the formula: length X width^2^ X 0.5. At the 28th day after injection, all mice were sacrificed and tumors were weighed and photographed.

### Co-immunoprecipitation assay and Western blot analysis

Co-immunoprecipitation assay and Western blot were performed as described [34]. Lysates were analyzed by immunoblot analysis using the specific antibodies as indicated in the text. Specific bands were detected with a horseradish peroxidase-conjugated antibody and revealed by an enhanced chemiluminescence (ECL) Western blot system (PerkinElmer, Waltham, MA).

### Chromatin immunoprecipitation (ChIP) and re-chromatin immunoprecipitation analysis

The ChIP assay was performed as described previously [11]. Briefly, the DNA-protein complex was eluted in elution buffer (1x TE buffer containing 1% SDS) with rotation at room temperature for 15 min, and immune complex crosslinking was reversed by heating to 65 °C overnight, followed by treating with 100 μg/ml of proteinase K at 50 °C for 1 h. DNA was extracted twice with phenol/chloroform and precipitated with ethanol. The DNA pellet was re-dissolved in H_2_O and subjected to Q-PCR amplification using specific primers for the *CPAP* promoter (sequence information provided in Additional file 1: Table S1). For re-ChIP, chromatin immunoprecipitates from the first ChIP were desalted by re-chip buffer (50 mM Hepes, 140 mM NaCl, 1 mM EDTA, 1% Triton X-100, 0.1% NaDOC, 0.1% SDS, and 0.5 mM PMSF), incubated with the second antibody or normal-serum control, and processed as above.

### Inducible expression of HBV and HBV infection

HepAD38/Tet-off cells were grown in DMEM with 0.3 μg/ml doxycycline, and the production of HBV was induced by removing doxycycline. HepG2-hNTCP-C4 cells were seeded at 5 × 10^5^ cells/well in a 6 cm collagen-coated plate. HBV derived from HepAD38/Tet-off cells was infected into HepG2-NTCP-C4 cells at 1000 genome equivalent (GEq)/cell; after 12 h infection, the media was replaced with fresh DMEM/F12 containing 2% FBS, 4% PEG 8000. The infected cells were washed three times with PBS at 0 day post infection (dpi), then incubated in fresh DMEM/F12 supplemented with 10% FBS. The cells were collected at 6 dpi for further analysis.

### Luciferase reporter assay

HCC cells were grown in 24-well plate and transfected with a mixture of different vectors as indicated in the figure legends using PolyJet transfection reagent (SignaGen, Rockville, MD) or Lipofectamine reagent (Invitrogen, Grand Island, NY). Luciferase activities were measured with Briteplus (PerkinElmer) after 24 or 48 h transfection. All experiments were repeated at least three independent times in triplicate.

### shRNA and RNA interference transfection

CPAP knockdown experiment is based on our previous reports [5, 34]. Briefly, *CPAP* siRNA duplex (5′-GGACUGACCUUGAAGAGAAUU-3′) and pSUPER-sh*CPAP* (5′-AAUGGAAUGCACGUGACGAUG-3′) were used by transfecting into HCC cells using Lipofectamine 2000 reagent according to the manufacturer’s instructions (Invitrogen).

### Subclone of GFP-HBx

HBx DNA obtined from Dr. Tasi [33] and subcloned into pEGFP-C1 vector by EcoRI restriction enzyme site.

### In situ proximity ligation assay (PLA)

HCC cells were grown on sterile cover slips, and after 24 h transfection with HA-CPAP/WT or MT [34] and GFP-HBx, cells were fixed in 3.7% formaldehyde for 10 min and then in situ PLA was performed according to the manufacturer’s instructions (Olink Bioscience, Uppsala, Sweden). Two primary antibodies derived from different species were used to recognize CPAP and GFP. Secondary antibodies were species-specific PLA probes. The interaction of proteins was amplified as distinct bright-red spots and detected using a fluorescence microscope (Personal DV Applied Precision, Issaquah, WA).

### RNA extraction and quantitative real-time PCR

Total RNA was extracted using TRIsure reagent (Bioline, London, UK). cDNA was synthesized with the High Capacity cDNA Reverse Transcription Kit (Applied Biosystems, Grand Island, NY). Quantitative real time-PCR assay to detect mRNA expression were conducted using iQ™ SYBR® Green Supermix (Bio-Rad, Hercules, CA). *Actin* was used as an internal control. Primers specific for human genes are described in Additional file 1: Table S1.

### Colony formation assay

For clonogenicity analysis, 2 × 10^3^ cells/well were seeded in 6-well plate and cultured in complete medium for 10~15 days. Colonies were fixed with formaldehyde and stained with 0.1% crystal violet.

### Cell proliferation assay

Cells (2 × 10^3^ cells/well) were seeded in 96-well plate and maintained in complete medium overnight. Cell proliferation was performed using the CCK-8 and BrdU incorporation at indicated times according to the manufacturer’s instructions.

### In vitro trans-well migration assay

Cells were resuspended in serum-free medium and 400 μl of cell suspension (1.5 × 10^5^ cells) were placed into the upper chamber (Millicell chambers, Millipore, Billerica, MA). Medium containing 10% FBS were added to the lower chamber. After 20 h of incubation, the cells remaining on upper chamber were removed, and the attached cells on the lower membrane were formaldehyde-fixed and stained with 0.1% crystal violet. The migration rate was quantified by counting the migrated cells in five random fields using an inverted microscope.

### The cancer genome atlas (TCGA) data set

Data from the TCGA data set was used to analyze the overall survival (OS) curves using SurvExpress biomarker validation tool [1].

### Statistical analysis

Statistical differences were assessed between experimental groups using two-tailed and un-paired Student’s t tests. *, *p* < 0.05; **, *p* < 0.01; ***, *p* < 0.001.

## Results

### Overexpression of CPAP in HBV-associated HCC

Our previous studies have showed that CPAP expression positively correlates with a poor prognosis in HBV-HCC [34]. To further assess the clinical significance of CPAP expression in HBV-HCC, we evaluated the association between CPAP and the major clinicopathological features of 132 HBV-HCCs (Additional file 1: Table S2). The results showed a significant correlation between high *CPAP* expression levels with the disease-free survival rate (DFS), aspartate transaminase (AST), alanine transaminase ALT, differentiation, tumor size, and AJCC stage (Table 1); and co-expression between overexpressed CPAP and HBx is positively correlated with DFS (Table 2).

**Table 1:** Associations between *CPAP* expression, clinical parameters and disease-free survival/ overall survival

Clinical parameters	*N*	Disease-free survival (years)				Overall survival (years)			
		Mean	95% CI		*p*-value	Mean	95% CI		*p*-value
*CPAP* expression									
< 1.5X	47	2.776	2.345	3.237	0.039*	3.858	3.376	4.340	0.521
≧1.5X	85	2.644	1.815	3.472		7.886	7.122	8.650	
Gender									
Male	102	2.379	2.039	2.720	0.922	7.616	6.837	8.394	0.413
Female	30	2.825	1.768	3.881		7.983	6.941	9.025	
Age (yrs)									
< 50	30	2.508	1.994	3.021	0.196	4.001	3.507	4.495	0.867
≧50	102	2.869	1.968	3.770		7.736	6.987	8.486	
Liver cirrhosis									
No	89	2.042	1.709	2.376	0.231	7.855	7.040	8.671	0.771
Yes	43	3.387	2.197	4.577		7.400	6.364	8.437	
AST/GOT (U/L)									
< 52	80	3.827	3.124	4.530	0.118	8.386	7.749	9.023	0.010*
≧52	52	2.189	1.658	2.720		6.532	5.278	7.787	
ALT/GPT (U/L)									
< 111	120	3.083	2.156	4.010	0.074	8.030	7.423	8.637	0.045*
≧111	12	1.706	0.822	2.591		2.648	1.935	3.361	
AFP (ng/ml)									
< 400	103	2.954	2.048	3.860	0.929	7.825	7.091	8.559	0.510
≧400	29	2.397	1.776	3.018		7.264	5.925	8.604	
Differentiation									
Well	25	2.583	1.891	3.274	0.063	7.957	7.254	8.661	0.009*
Moderate	91	3.850	3.176	4.525		7.754	6.950	8.559	
Poor	16	1.207	0.764	3.900		3.399	2.227	4.571	
Tumor size (cm)									
< 5	87	3.536	2.455	4.617	0.000*	8.346	7.822	8.870	0.000*
≧5	45	1.529	1.086	1.973		6.007	4.500	7.513	
Tumor number									
Single	107	3.034	2.092	3.976	0.921	7.764	7.033	8.495	0.631
> 1	25	2.155	1.572	2.739		3.227	2.735	3.718	
Vascular invasion									
Absence	94	3.014	2.077	3.951	0.601	8.168	7.468	8.869	0.057
Presence	38	2.110	1.626	2.593		6.764	5.532	7.997	
AJCC Stage									
I&II	108	3.451	2.402	4.501	0.000*	8.238	7.626	8.850	0.001*
III	24	0.910	0.526	1.295		3.074	2.281	3.867	

*, statistically significant

**Table 2:** Associations between CPAP expression, HBx expression, and disease-free survival/overall survival (Kaplan-Meier analysis)

Clinical parameters	*N*	Disease-free survival (years)				Overall survival (years)			
		Mean	95% CI		*p*-value	Mean	95% CI		*p*-value
CPAP expression									
< 1.5X	47	2.776	2.315	3.237	0.039*	3.858	3.376	4.340	0.521
≧1.5X	85	2.644	1.815	3.472		7.886	7.122	8.650	
HBx expression									
< 1X	97	3.096	2.125	4.066	0.527	7.801	7.056	8.546	0.929
≧1X	35	1.949	1.473	2.424		7.425	6.194	8.657	
CPAP+HBx expression									
CPAP< 1.5X, HBx < 1X	34	2.845	2.325	3.364	0.045*	3.583	3.104	4.063	0.537
CPAP≧1.5X, HBx≧1X	22	1.458	1.061	1.855		7.830	6.391	9.270	

*, statistically significant

### HBx transcriptionally up-regulates CPAP by interacting with CREB

**Fig. 1:**
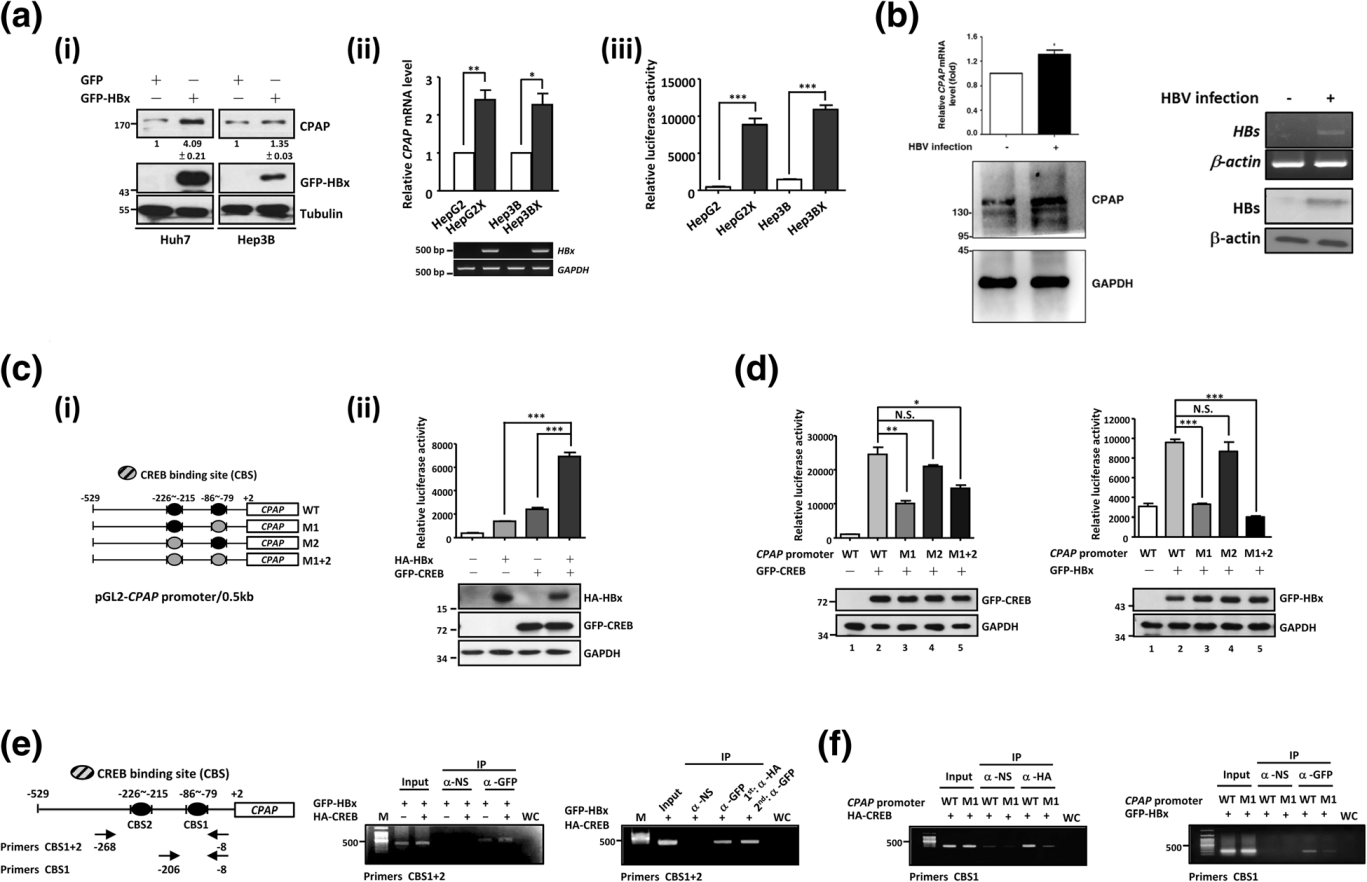
CREB is crucial for HBx to associate with *CPAP* promoter and to enhance the *CPAP* promoter activity. **a** (i) Huh7 or Hep3B cells with GFP or GFP-HBx expression were collected to detect the expression of CPAP and GFP-HBx by Western blot analysis. The mRNA level (ii) and promoter activity (iii) of *CPAP* in HepG2, HepG2X, Hep3B and Hep3BX cells were determined by RT-qPCR (for *CPAP* mRNA) or RT-PCR (for *HBx* mRNA) and reporter assay, respectively. The expression levels of endogenous CPAP are indicated as ratio (CPAP/Tubulin). The mean ± SD was obtained from triplex Q-PCR, and three independent experiments were performed. Error bars of reporter assay represent the mean ± SD of three independent experiments, each performed in triplicate. **b** The expressions of CPAP (left) and HBV surface antigen (HBs) (right) were determined by Q-PCR or RT-PCR and Western blot analysis in HBV-infected (+) or no infected (−) HepG2-NTCP-C4 cells [14]. **c** (i) A schematic representation of the *CPAP* proximal promoter (pGL2-*CPAP* promoter/0.5 kb) is shown. Two potential CREB binding sites (CBSs) are showed in black ovals (WT), and the promoter with either one or both CBSs mutations is presented by gray color (M1, M2, or M1 + M2). (ii) *CPAP* promoter activity was examined in Huh7 cells with GFP-CREB or/and HA-HBx expression by reporter assay. **d** pGL2-*CPAP*/WT, M1, M2 or M1 + 2 was transiently transfected into Huh7 cells with GFP-CREB (left) or GFP-HBx (right), and then, the luciferase activity was analyzed. Error bars represent the mean ± SD of three independent experiments, each performed in triplicate. *, *p* < 0.05; **, *p* < 0.01; ***, *p* < 0.001; N.S., no significance. **e** (Left) Primers used in chromatin immunoprecipitation (ChIP) and re-ChIP assay of the *CPAP* promoter are shown. The ChIP (middle) and re-ChIP (right) assay were performed in GFP-HBx- and HA-CREB-expressing Huh7 cells by anti-GFP, anti-HA antibody and normal serum control. **f** pGL2-*CPAP*/WT or M1 and HA-CREB (left) or GFP-HBx (right) were co-transfected into Huh7 cells, and then analyzed by ChIP assay using anti-HA or anti-GFP antibody as described in (**e**). WC: water control

By Western blot analysis, we found that HBx can increase CPAP expression in HCC cells (Fig. 1A-i). The expression level of *CPAP* mRNA was higher in HBx stable expression cell lines Hep3BX or HepG2X than Hep3B or HepG2 (Fig. 1A-ii). The *CPAP* promoter assay further demonstrated that HBx transcriptionally up-regulates the *CPAP* promoter (Fig. 1A-iii, and Additional file 2: Figure S1). The same result was obtained from HBV-infected HCC or HBV genome-expressing HCC cells [14]. Expression of *CPAP* mRNA and protein was increased in HBV-infected (Fig. 1B) or HBV genome-expressed HCC (Additional file 2: Figure S2). To identify the specific transcription factors involved in HBx-enhanced transcriptional activation of *CPAP*, TFSEARCH (http://cbrc3.cbrc.jp/papia/howtouse/howtouse_tfsearch.html) was used to evaluate the *CPAP* promoter, and two potential CREB binding sites (CBSs) were found (Fig. 1C-i, − 226 to − 215 bp and − 86 to − 79 bp). *CPAP* promoter activity was significantly decreased when CREB binding site 1 was mutated (M1), whereas a mutation in CREB binding site 2 (M2) only moderately decreased the promoter activity compared with the wild type control (Additional file 2: Figure S3). Given that HBx has been shown to interact with CREB-binding protein/p300 to mediate CREB-dependent transcription [20, 37], we assumed that HBx might activate the *CPAP* promoter through the interaction with CREB. As anticipated, overexpression of CREB or HBx increased *CPAP* promoter activity, which was further enhanced by co-expression of HBx and CREB (Fig. 1C-ii and Additional file 2: Figure S4A). Overexpression of the CREB dominant-negative (DN) mutant had a smaller effect on *CPAP* promoter activity (Additional file 2: Figure S4B and C); knocked-down expression of *CREB* led to decreased *CPAP* promoter activity (Additional file 2: Figure S5A and B). Either HBx or CREB can induce wild type *CPAP* promoter activity but failed to affect *CPAP*/M1 or M1 + 2 promoter activities (Fig. 1D and Additional file 2: Figure S4D). Overexpression of HBx retained the ability to activate *CPAP* promoter in *CREB* knocked-down cells (Additional file 2: Figure S5C), while the CREB expression is still maintained in a low level (Additional file 2: Figure S5A).

We further investigated whether the transcriptional up-regulation of *CPAP* by HBx was due to HBx interacting with CREB and binding to the *CPAP* promoter. ChIP assay confirmed that overexpressed CREB can enhance the association of HBx with the *CPAP* promoter, re-Chip assay further demonstrated that CREB can form a complex with HBx to bind to the *CPAP* promoter (Fig. 1E). By contrast, CREB and HBx did not associate with the *CPAP*/M1 promoter (Fig. 1F). These results indicated that the *CPAP* promoter − 86 to − 79 bp is a *cis*-regulatory element for HBx-mediated transcriptional activation; HBx enhances *CPAP* expression by binding onto the *CPAP* promoter through the association with CREB.

### CPAP and HBx cooperatively enhance NF-κB activation

**Fig. 2:**
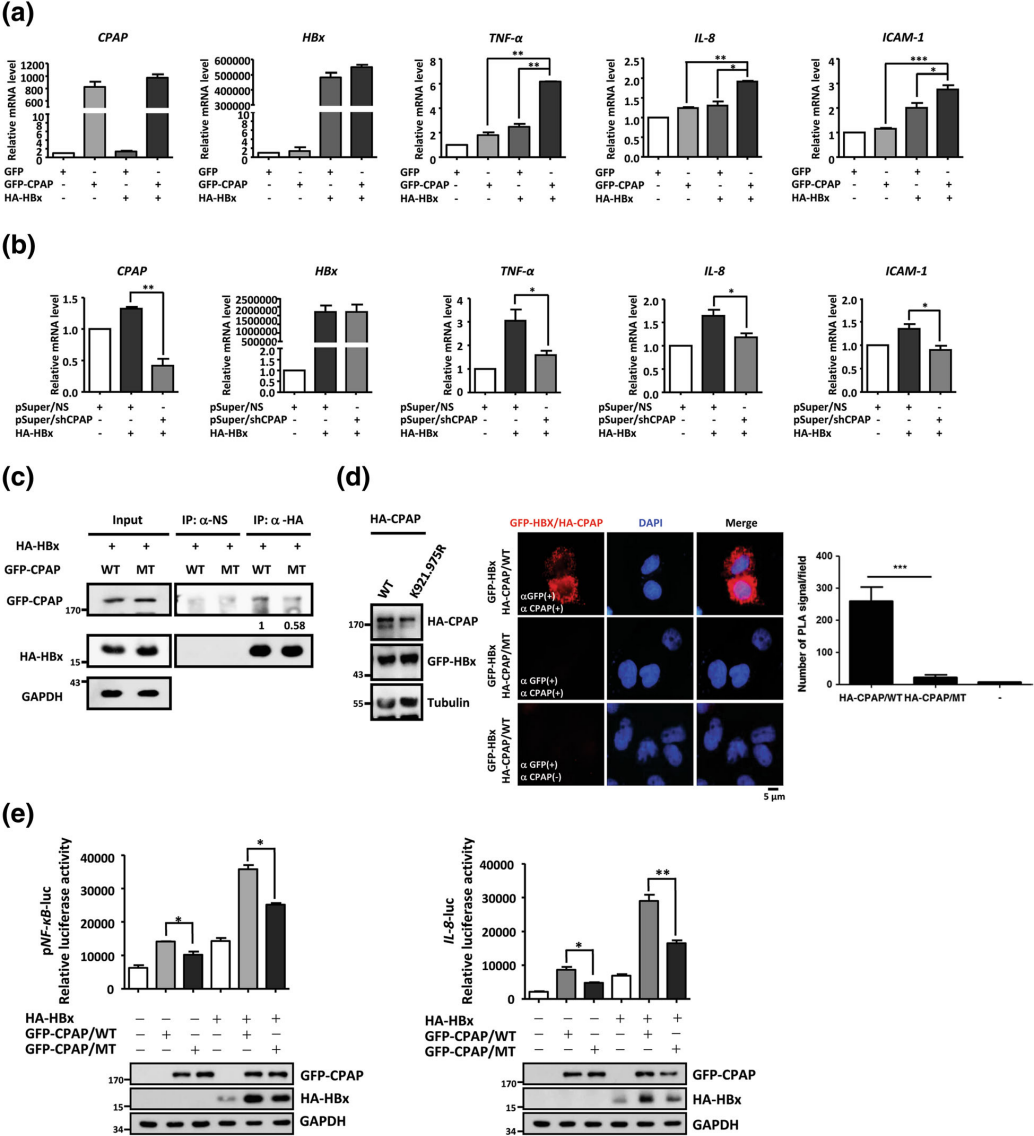
CPAP interacts and cooperates with HBx to enhance NF-κB target genes expression. **a-b** The mRNA level of NF-κB-mediated target genes (*TNF-α*, *IL-8*, *ICAM-1*) were detected by Q-PCR in Huh7 cells with co-expressed HA-HBx and GFP-CPAP (A) or knockdown of CPAP (B, pSuper-CPAP). Data present three independent experiments, and each performed in triplicate (*, *p* < 0.05; **, *p* < 0.01; ***, *p* < 0.001). **c** Immunoprecipitation (IP) assay was performed in total cell lysates from Huh7 with GFP-CPAP/WT or GFP-CPAP/MT and HA-HBx using anti-HA antibody. The immunoprecipitates were further detected by Western blot analysis by antibodies as indicated. The interaction ability between GFP-CPAP and HA-HBx is indicated as ratio. **d** The interaction between CPAP and HBx was determined by in situ PLA using anti-CPAP and anti-GFP antibodies (CPAP+, GFP+). The red spots represent interacting complexes of CPAP and HBx. Cells stained with anti-GFP antibody only (CPAP-, GFP+) were used as a negative control. The nuclei were stained with DAPI (blue). The quantitative results of in situ PLA was shown. **e** NF-κB-responsive transcriptional activity (left) and *IL-8* promoter activity (right) in cells with GFP-CPAP/WT or GFP-CPAP/MT combined with or without HA-HBx were determined by reporter assay. The error bars represent the mean ± SD of three independent experiments, each performed in triplicate (*, *p* < 0.05; **, *p* < 0.01)

It has been well recognized that NF-κB is a transcription factor regulated by HBx, and contributes to tumorigenesis by activating the expression of several genes involved in the immune response, inflammation, cell proliferation and the prevention of apoptosis [22]. On the other hand, it has been reported that CPAP is essential for the transcriptional activation of NF-κB and synergistically increases HBx-mediated NF-κB activation [13, 34]. Considering the data from clinical specimens of HBV-HCCs and the critical role of CPAP in NF-κB activation, we investigated the molecular functions of CPAP and HBx in NF-κB signaling. The results showed that the either overexpression of CPAP or HBx both increases the transcriptional activity of NF-κB, and the co-expression of CPAP and HBx induced a substantial increase in NF-κB-responsive transcriptional activity (Additional file 2: Figure S6A, left panel). Knockdown of *CPAP* by siRNA diminished the HBx-induced NF-κB activation (Additional file 2: Figure S6A, right panel), confirming that CPAP is essential for HBx-enhanced NF-κB activation. The same effects were found using the *IL-8* promoter, activation of which is dependent on NF-κB activity (Additional file 2: Figure S6B and C). Individual expression or co-expression of CPAP and HBx increased the expression of the NF-κB downstream targets *TNF-α*, *IL-8* and *ICAM-1* (Fig. 2a), whereas *CPAP* knockdown reduced the induction of NF-κB-mediated gene expression by HBx (Fig. 2b).

To further investigate how CPAP and HBx cooperate to enhance NF-κB activity, we first checked the association between CPAP and HBx in the NF-κB pathway. Co-immunoprecipitation analysis and in situ PLA indicated that CPAP interacts with HBx (Fig. 2 c, d and Additional file 2: Figure S7A). Our previous report indicated that TNF-α-mediated SUMO-1 modification of CPAP is important for NF-κB activation [34]. Here, we investigated whether SUMO modification of CPAP is required for HBx-induced NF-κB activation. The results showed that SUMO-deficient CPAP did not interaction with HBx (Fig. 2 c, d and Additional file 2: Figure S7A), and decreased the HBx-enhanced NF-κB transcriptional activity (Fig. 2e). Furthermore, the interaction between CPAP and HBx was increased upon TNF-stimulation (Additional file 2: Figure S7B). These findings suggest that SUMO modification is essential for CPAP to associate with HBx and cooperatively enhance NF-κB activity.

### SUMO modification is required for CPAP to promote HCC proliferation and tumorigenicity

Next, we examined the role of CPAP in HCC development. HepG2 cells that stably expressed GFP-CPAP/WT exhibited a higher proliferative rate and increased colony formation compared with GFP-CPAP/MT and control cells (Additional file 2: Figure S8A). Moreover, *NF-κB/p65* knockdown decreases CPAP-enhanced colony formation in Hep3B cells (Additional file 2: Figure S9), suggesting that the CPAP-enhanced proliferation of HCC is dependent on NF-κB activity. To further determine the effect of CPAP on hepatoma cell growth in vivo, GFP-CPAP/WT or GFP-CPAP/DM stably expressed HCC cells were subcutaneously injected into NOD/SCID mice. The results showed that overexpression of CPAP/WT significantly increased tumor growth compared with CPAP/DM (Additional file 2: Figures S8B and S10). These results suggest that CPAP promotes HCC growth both in vitro and in vivo.

### CPAP and HBx cooperatively enhance HCC proliferation, migration and tumorigenesis

**Fig. 3:**
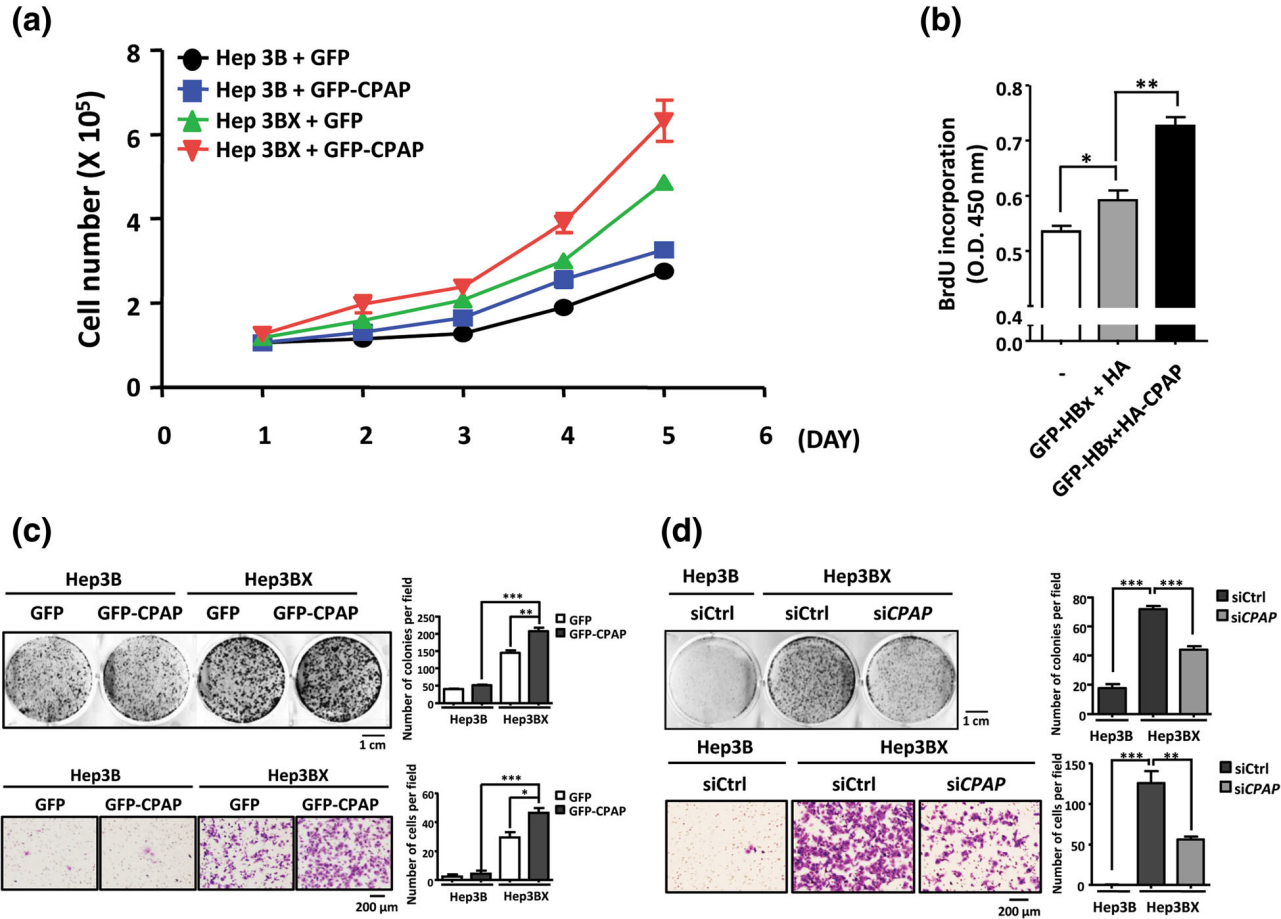
CPAP expression increases HBx-induced cell growth and proliferation of HCC cells. **a** Proliferation of Hep3B and Hep3BX cells with ectopic expressed GFP or GFP-CPAP were determined by counting cell numbers every 24 h under monolayer culture conditions. **b** Cell proliferation was examined by BrdU incorporation assay in Hep3B cells with GFP-HBx/HA or GFP-HBx/HA-CPAP expression. The mean ± SD was obtained from three independent experiments (*, *p* < 0.05; **, *p* < 0.01). **c** Hep3B and Hep3BX cells with GFP or GFP-CPAP expression were subjected to colony-formation assay (upper) and in vitro trans-well migration assay (lower). **d** Hep3BX cells with *CPAP* siRNA (si*CPAP*) or control siRNA (siCtrl) were plated for colony-formation assay (upper) and in vitro trans-well migration assay (lower) as mentioned above (**c**). Hep3B with control siRNA (siCtrl) was used as a control. The number of cells was counted in five randomly selected fields. The data is presented as the mean ± SEM in three independent experiments (*, *p* < 0.05; **, *p* < 0.01; ***, *p* < 0.001)

Next, we evaluated the contribution of CPAP in HBx-mediated hepatocarcinogenesis by investigating the effects of CPAP on the tumorigenic properties of HCC cells. The results indicated that Hep3B cells expressing either CPAP or HBx showed a higher proliferative rate and colony number than Hep3B cells; co-expression of CPAP significantly increased HBx-enhanced cell growth (Fig. 3 a, b and c/upper panel). Moreover, expression of CPAP in Hep3BX cells enhanced HBx-induced cell migration ability (~ 1.5 fold, Fig. 3c/lower panel). To further validate the effect of CPAP in these observations, CPAP was knocked down in Hep3BX cells, and the results showed an impairment of HBx-induced cell growth and migration (Fig. 3 d). These results support that CPAP is crucial for HBx-induced tumorigenesis in HCC.

### CPAP is required for maintaining TNF-α-mediated HBx protein stability through enhancing the NF-κB activity

**Fig. 4:**
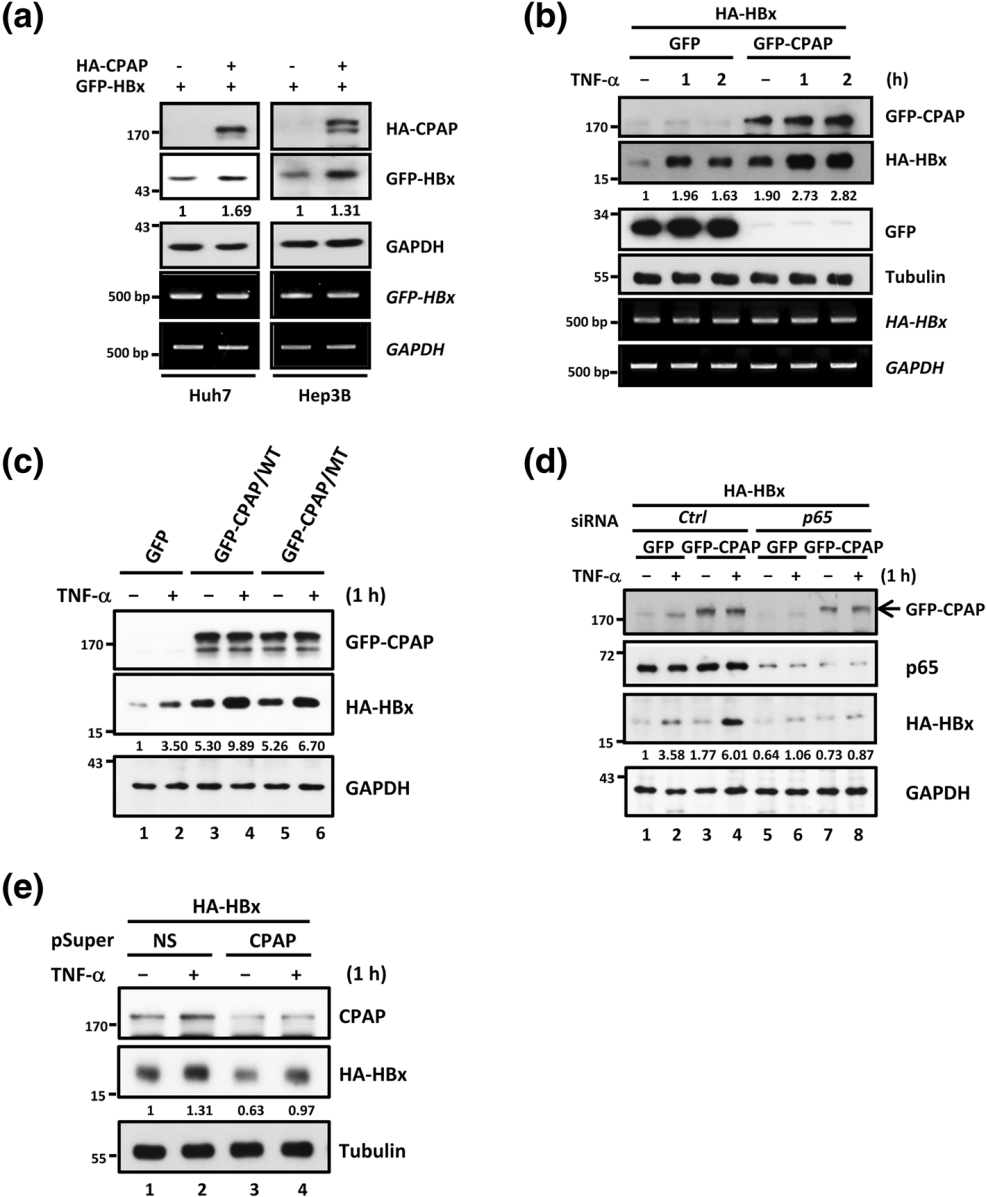
CPAP augments TNF-α-mediated HBx stabilization in a NF-κB-dependent manner. **a** HCC cells with HA-CPAP or GFP-HBx were analyzed by Western blot analysis and RT-PCR. **b** Huh7 cells with HA-HBx and GFP or GFP-CPAP were treated with TNF-α (10 ng/ml) for 0, 1, and 2 h. The expression level of GFP, GFP-CPAP, and HA-HBx was determined by Western blot and/or RT-PCR as described in Fig. 1a. **c** Western blot analysis of Huh7 cells with GFP-CPAP/WT expression increased HA-HBx stability upon TNF-α treatment (10 ng/ml, 1 h) compared with GFP-CPAP/MT or GFP control. **d** Huh7 cells were transfected with non-targeting siRNA (*Ctrl*) or *NF-κB/p65* siRNA (*p65*) for 24 h. The protein levels were examined by Western blot analysis after further transfection of HA-HBx and GFP or GFP-CPAP for an additional 24 h with TNF-α treatment for 1 h. **e** Huh7 cells were transiently transfected with HA-HBx and pSuper/CPAP or pSuper/NS control for 24 h. Protein expression of CPAP and HA-HBx was determined after treating cells with TNF-α for 1 h. The expression levels of HA-HBx are indicated as ratio, and three independent experiments were performed

Previous study demonstrated that TNF-α can induce a notable accumulation of HBx by increasing protein stability due to reduced proteasomal degradation through NF-κB signaling. Interestingly, we found that the protein expression, but not the mRNA, of GFP-HBx was increased in HA-CPAP-overexpressing cells (Fig. 4a), indicating that the prolonged protein stability of GFP-HBx may under the regulation of HA-CPAP. Since CPAP is a co-activator of IKK-mediated NF-κB activation in response to TNF-α treatment [34], we further clarified whether CPAP can increase the protein stability of HBx through enhancing TNF-α-induced NF-κB activation. The results showed that the highest level of HBx protein was observed 1 h after TNF-α treatment, and then slowly decreased in a time-dependent manner (Additional file 2: Figure S11A). Overexpressed CPAP significantly augmented HBx protein expression but not mRNA expression in response to TNF-α treatment (Fig. 4b). To verify whether the accumulation of HBx protein induced by CPAP is associated with the increased stability of HBx, HCC cells with HA-CPAP and GFP-HBx expression were treated with the protein synthesis inhibitor cycloheximide, and the level of GFP-HBx in the absence or presence of TNF-α was determined at various time points. The results showed that CPAP significantly increased TNF-α-induced HBx stabilization (Additional file 2: Figure S11B).

Our previous study indicated that SUMOylated CPAP is important for TNF-α-mediated NF-κB activation [34], therefore, we investigated whether SUMOylation of CPAP is necessary for HBx protein stability. The results showed that CPAP/WT increased HBx protein expression, whereas the CPAP SUMO-deficient mutant had only a minor effect on maintaining HBx protein expression even in the presence of TNF-α (Fig. 4c, compare lanes 4 and 6). Next, we knocked down *NF-κB/p65* and evaluated the effect of CPAP-enhanced TNF-α-induced HBx stabilization. The results showed that the expression of HA-HBx was markedly reduced in *NF-κB/p65* knocked down cells even in the presence of TNF-α (Fig. 4d, compare lanes 2 and 6). Moreover, the overexpression of CPAP could not enhance TNF-α-induced HBx stabilization in *p65*-knocked down cells (Fig. 4d, compare lanes 4 and 8). In addition, after the knockdown of *CPAP*, HBx expression was decreased regardless of TNF-α treatment (Fig. 4e, compare lanes 2 and 4). These results suggest that CPAP is required for TNF-α-induced HBx stabilization.

### Overexpressed CREB/CPAP positively correlates with a poor survival rate in HBx-positive HCC

**Fig. 5:**
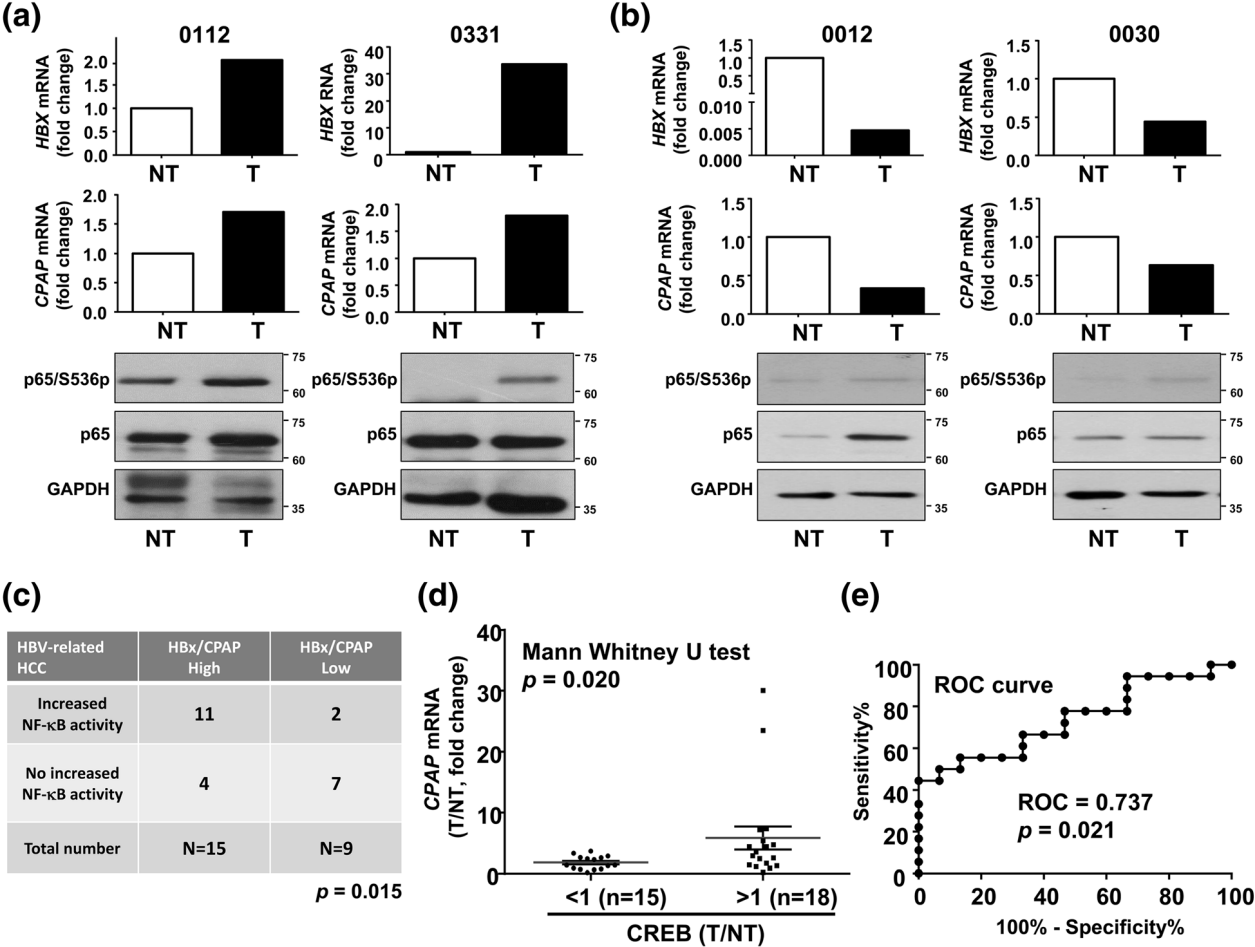
Augmented NF-κB activity in CPAP-overexpressed HBx positive HCC. **a-b** HCC tissues with (**a**) or without (**b**) overexpressed CPAP and HBx were collected for Western blot analysis to detect the expression level (p65) and the activation status (p65/S536p) of NF-κB. GAPDH was used as a loading control. The expression levels of *CPAP* and *HBx* mRNAs in HCC were detected by Q-PCR, normalized by *actin* mRNA (NT, non-tumor part; T, tumor part). Total of 24 HCC tissues with (*N* = 15) or without (*N* = 9) overexpressed HBx/CPAP were analyzed, and two representative cases for both (**a**) and (**b**) are shown. **c** The correlation between CPAP/HBx expression and activated NF-κB was found to be statistically significant by Chi-square analysis; *p* = 0.015. **d** The expression level of *CREB* mRNA is determined in HBx-positive HCC by Q-PCR. A total of 33 specimens were enrolled, and the correlation between overexpressed CREB (T/NT > 1) and CPAP expression was analyzed by Mann Whitney U test (two-tailed); *p* = 0.020. **e** ROC (receiver operating characteristic curve) analysis of overexpressed CREB and CPAP in HBx-positive HCC from (D) (*p* = 0.021)

In order to give insight into the clinical impact of the interaction between HBx and CPAP, we checked the activation status of NF-κB in CPAP-overexpressing HBx-positive HCC. The result indicated that HCC tissues with overexpressed HBx and CPAP has an increased activation of NF-κB (Fig. 5a), whereas HCC tissues without HBx and CPAP overexpression present no enhanced NF-κB activity (Fig. 5b). The correlation between enhanced NF-κB phosphorylation with overexpressed HBx and CPAP is statistically significant (Fig. 5c). The correlation between overexpressed HBx and CPAP with activated NF-κB is positively correlated with DFS (*p* = 0.0086) in HCC patients (Additional file 2: Figure S12). Overexpressed CREB is positively correlated with an augmented CPAP level in HBx-positive HCC (Fig. 5d, e). Importantly, overexpressed CPAP/CREB showed a lower DFS than instances with a lower expression level of CPAP/CREB (Additional file 2: Figure S13A). We also analyzed the TCGA data set to check the correlation between the overall survival rate (OS) and overexpressed CPAP/CREB using SurvExpress biomarker validation tool [1], and the results indicated that an increased expression of *CREB* and *CPAP* mRNAs exists in the high-risk group with a lower survival rate (Additional file 2: Figure S13B).

## Discussion

**Fig. 6:**
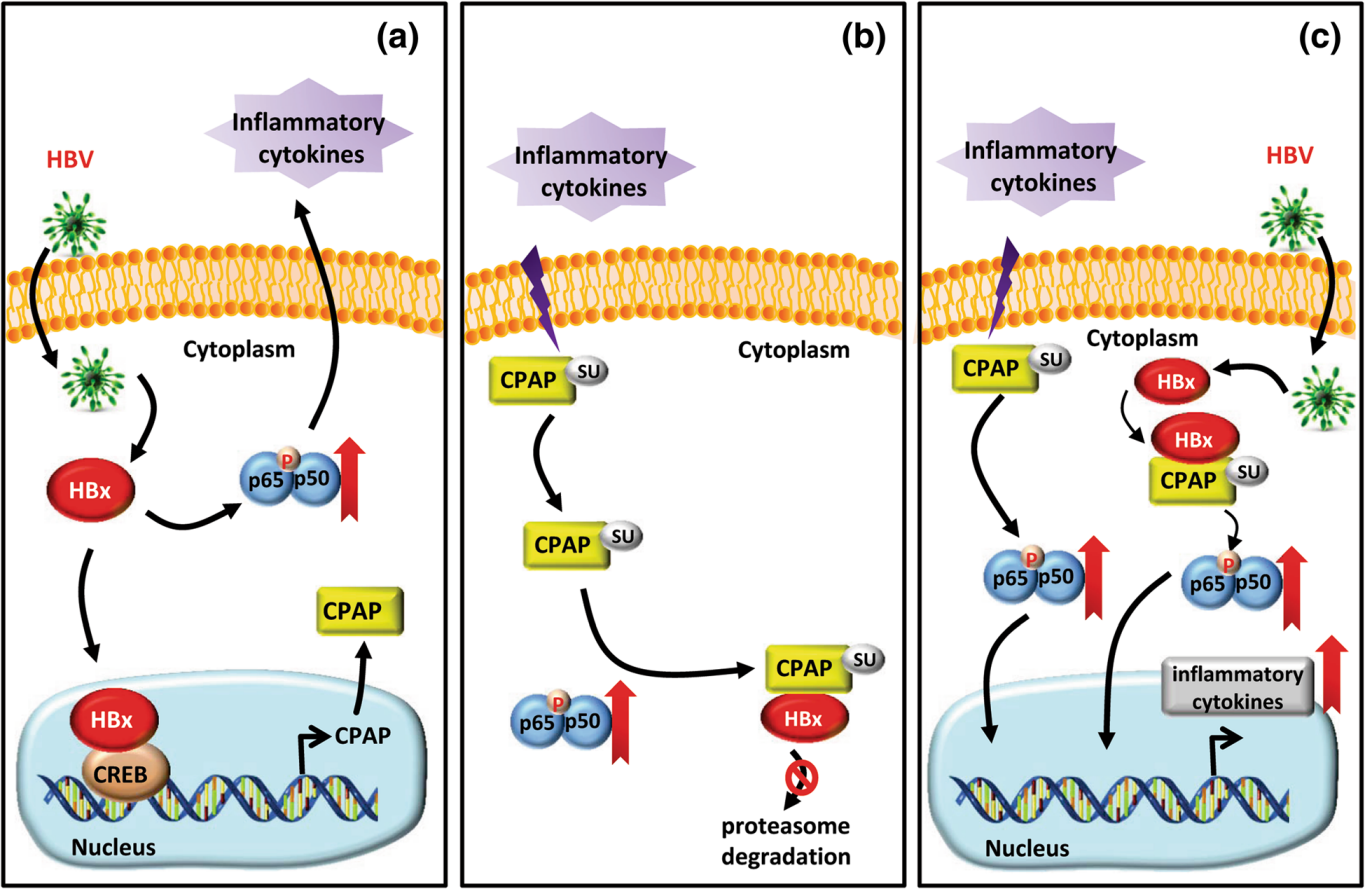
Model of the CPAP/HBx interaction in hepatocarcinogenesis. HBV infection triggers immune responses and later develops into chronic liver diseases, such as hepatitis, fibrosis or cirrhosis. The further production of inflammatory cytokines induced by HBx can modulate the SUMO modification of CPAP and subsequently enhance IKK-mediated NF-κB activation. **a** In this study, we demonstrate that HBx associates with the transcription factor CREB to up-regulate *CPAP* transcription. HBx promotes NF-κB activity to enhance the production of inflammatory cytokines. **b** Meanwhile, inflammatory cytokines increases HBx stabilization, which is enhanced by CPAP expression and dependent on NF-κB activity. **c** CPAP interacts with HBx to further promote HBx-induced NF-κB activation and hepatocellular transformation, finally resulting in hepatocarcinogenesis

HBV integrates its viral DNA into the host cellular DNA to disrupt or promote the expression of cellular genes which then influences cell growth and differentiation. HBV infection is a crucial factor in chronic or acute hepatitis and contributes to the development of HCC [31]. Our study proposes a novel model of the relationship between CPAP and HBx in HCC development (Fig. 6). Chronic HBV infection in liver cells triggers host immune responses and results in hepatitis, and the release of inflammatory cytokines can induce SUMO modification of CPAP, as previously shown [34]. HBx transcriptionally up-regulates the expression of *CPAP* by interacting with CREB (Fig. 6a). Meanwhile, SUMOylated CPAP further enhances NF-κB activation and inflammatory cytokine expression; and SUMO modification of CPAP leads to its further association with HBx and increases TNF-α-induced HBx protein stabilization in an NF-κB/p65-dependent manner (Fig. 6b). The reciprocal regulation of CPAP and HBx cooperatively increases NF-κB activity and finally contributes to hepatocarcinogenesis (Fig. 6c).

Previous studies have shown that HBx is expressed in human liver specimens with high necroinflammatory activity [19]. The chemokine-dependent recruitment of inflammatory cells by HBx involves the activated NF-κB pathway. The NF-κB-induced expression of cytokines, including interleukin-1 (IL-1), IL-6, TNF-α, and interferon-γ (INF-γ), plays an important role in inflammatory processes depending on the duration of inflammation. Remarkably, IL-6 and TNF-α can activate NF-κB in turn to increase the stability of HBx protein in an NF-κB-dependent manner [27]. Therefore, it is postulated that positive feedback between HBx and proinflammatory cytokines produced by NF-κB in the microenvironment in the liver leads to hepatic inflammation in chronic hepatitis B. It does not known whether additional proteins can be recruited to enhance HBx stability. In the present study, we demonstrated that CPAP contributes to the protein stability of HBx through increased NF-κB activity (Fig. 4). CPAP has been previously identified as a co-activator of NF-κB and as a scaffold protein for the association of IKKs and NF-κB [13, 34]. This report indicates that CPAP enhances NF-κB activation to increase the expression of inflammatory cytokines, further resulting in TNF-α-induced HBx stabilization. Moreover, if the CPAP/NF-κB pathway can be disrupted, the HBV-infected liver cells are able to reduce HBx-mediated HCC development. As mentioned in previous studies, C-terminal modification of CPAP with SUMO is stimulated by TNF-α and occurs close to the NF-κB/p65-interacting domain of CPAP [13, 34]. SUMO modification is also required for the interaction of CPAP and HBx, and for the enhanced effect of CPAP on TNF-α-induced HBx stabilization [34]. It remains to be elucidated whether the complex of SUMOylated CPAP and NF-κB/p65 can prevent the association of HBx with proteasome subunits to increase HBx protein stability.

## Conclusion

Here, we demonstrate a novel regulatory mechanism between CPAP and HBx in inflammation-related HCC development. The HBx/inflammatory cytokines/CPAP regulatory loop resulted in marked NF-κB activation in HBV-associated HCC, which provides a microenvironment for tumor development. Additionally, overexpressed CREB/CPAP indicated a poor prognostic value in HBV-associated HCC. Taken together, our findings provide an alternative therapeutic target in the NF-κB pathway to reduce the immunodeficiency caused by NF-κB inhibition, which leads to a novel therapeutic strategy for HBV-association HCC and other chronic inflammatory diseases.

## Additional files

Additional file 1: **Table S1.** Primers used in PCR analysis. **Table S2.** Clinical parameters of the patients with HBV-related HCC who were included in this study. (PDF 81 kb)

Additional file 2: **Figure S1.** HBx increases CPAP promoter activity in HCC cell lines. **Figure S2.** Expression of CPAP is increased in HBV genome-expressing HCC cells. **Figure S3.** The CREB binding site is crucial for *CPAP* promoter activity. **Figure S4.** CREB is essential for HBx-mediated *CPAP* promoter activity. **Figure S5.** Evaluation of CPAP promoter activity in sh*CREB* transfected Hep3B cells. **Figure S6.** CPAP is involved in HBx-induced transcriptional activation of NF-κB. **Figure S7.** The interaction between CPAP and HBx is increased upon TNF-α treatment. **Figure S8.** CPAP promotes proliferation, colony formation, and tumorigenicity of HCC. **Figure S9.** NF-κB/p65 is essential for CPAP-mediated colony formation of HCC cells. **Figure S10.** Overexpression of CPAP/WT significantly increased tumor growth in a xenograft animal model. **Figure S11.** CPAP increases TNF-α-mediated HBx protein stabilization. **Figure S12.** The clinical outcome of overexpressed CPAP, HBx and activated NF-κB (p65) in HCC. **Figure S13.** Co-overexpression of CPAP and CREB is positively correlated with a poor disease-free survival rate in HBx-positive HCC. (PDF 1009 kb)

## Abbreviations

ALT: Alanine transaminase
AST: Aspartate transaminase
BrdU: Bromodeoxyuridine (5-bromo-2′-deoxyuridine)
ChIP: Chromatin immunoprecipitation
CPAP: Centrosomal P4.1-associated protein
CREB: cAMP response element binding protein
GAPDH: Glyceraldehyde 3-phosphate dehydrogenase
GFP: Green fluorescent protein
HBV: hepatitis B virus
HBx: X protein of HBV
HCC: Hepatocellular carcinoma
ICAM-1: Intercellular adhesion molecule 1
IL-8: Interleukin-8
NF-κB: Nuclear factor kappa light chain enhancer of activated B cells
PLA: Proximity ligation assay
Q-PCR: Quantitative-PCR
TNFα: Tumor necrosis factor alpha
