# Supplementary material for: Hepatitis B virus X protein (HBx) enhances centrosomal P4.1-associated protein (CPAP) expression to promote hepatocarcinogenesis
Source: J Biomed Sci. 2019 Jun 6;26:44. doi: 10.1186/s12929-019-0534-9 (PMC6551916; doi:10.1186/s12929-019-0534-9)
Supplement: Supplementary file 1 — Table S1. Primers used in PCR analysis. Table S2. Clinical parameters of the patients with HBV-related HCC who were included in this study. (PDF 81 kb) [file 12929_2019_534_MOESM1_ESM.pdf]

Additional file 1

Table S1. Primers used in PCR analysis.

Gene	Forward primer (5’→3’)	Reverse primer (5’→3’)
**Q-PCR**		
CPAP	AGCCCTCGAGATCCTCATCCCT	TAGCATGTCTGCGGCGTCCC
qHBx	GCGCGGGACGTCCTTTGTCT	GTCGGCCGGAACGGCAGATG
TNF-α	CTTCTCCTTCCTGATCGTGG	GCTGGTTATCTCTCAGCTCCA
IL-8	GACAAGAGCCAGGAAGAAACC	CTTTAGCACTCCTTGGCAAAA
ICAM-1	GGCCTCAGTCAGTGTGA	AACCCCATTCAGCGTCA
Actin	CTGGACTTCGAGCAAGAGATG	TGATGGAGTTGAAGGTAGTTTCG
**RT-PCR**		
HBx	AAGCTTGCTGCTCGGGTGTGCTGCCAA	GGTACCGGCAGAGGTGAAAAAGTTGCA
GAPDH	CCCACTCCTCCACCTTTGAC	TCTCTCTTCCTCTTGTGCTCTTG
**ChIP aaasy**		
M1+2	CGGTTGGGCTCTATGTTAGC	GTGGGAAGTGAGGATCTCACC
M1	CTCCGTTAGGCGTTCCCTTTAG	GTGGGAAGTGAGGATCTCACC

Table S2. Clinical parameters of the patients with HBV-related HCC who were included in this study.

Clinical parameters	CPAP low (< 1.5X)	CPAP high (≧1.5X)
	N (%)	*N* (%)
Total number of patients	47	85
Gender		
Male	40 (30.3)	62 (47.0)
Female	7 (5.3)	23 (17.4)
Age (yrs)		
< 50	10 (7.6)	20 (15.2)
≧50	37 (28.0)	65 (49.2)
Liver cirrhosis		
No	34 (25.8)	55 (41.7)
Yes	13 (9.8)	30 (22.7)
AST/GOT (U/L)		
< 52	27 (20.5)	53 (40.2)
≧52	20 (15.2)	32 (24.2)
ALT/GPT (U/L)		
< 111	43 (32.6)	77 (58.3)
≧111	4 (3.0)	8 (6.1)
AFP (ng/ml)		
< 400	39 (29.5)	64 (48.5)
≧400	8 (6.1)	21 (15.9)
Differentiation		
Well	8 (6.1)	17 (12.9)
Moderate	33 (25.0)	58 (43.9)
Poor	6 (4.5)	10 (7.6)
Tumor size (cm)		
< 5	30 (22.7)	57 (43.2)
≧5	17 (12.9)	28 (21.2)
Tumor number		
Single	36 (27.3)	71 (53.8)
> 1	11 (8.3)	14 (10.6)
Vascular invasion		
Absence	39 (29.5)	55 (41.7)
Presence	8 (6.1)	30 (22.7)
AJCC Stage		

I & II	41 (31.1)	67 (50.8)
III	6 (4.5)	18 (13.6)
Recurrence		
No	34 (25.8)	43 (32.6)
Yes	13 (9.8)	42 (31.8)
