# Supplementary material for: Hepatitis B virus X protein (HBx) enhances centrosomal P4.1-associated protein (CPAP) expression to promote hepatocarcinogenesis
Source: J Biomed Sci. 2019 Jun 6;26:44. doi: 10.1186/s12929-019-0534-9 (PMC6551916; doi:10.1186/s12929-019-0534-9)

Figure S1

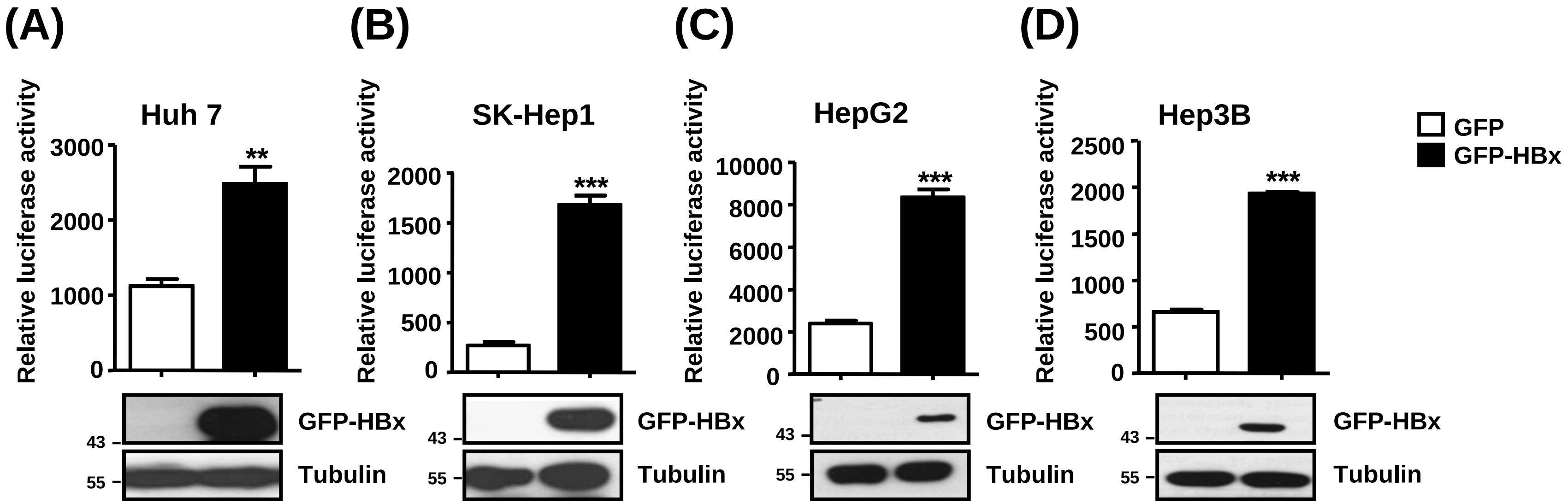

(A)
Huh 7
Relative luciferase activity
3000
2000
1000
0
**
43
55
GFP-HBx
Tubulin
(B)
SK-Hep1
Relative luciferase activity
2000
1500
1000
500
0
***
43
55
GFP-HBx
Tubulin
(C)
HepG2
Relative luciferase activity
10000
8000
6000
4000
2000
0
***
43
55
GFP-HBx
Tubulin
(D)
Hep3B
Relative luciferase activity
2500
2000
1500
1000
500
0
***
43
55
GFP-HBx
Tubulin
GFP
GFP-HBx

Figure S2

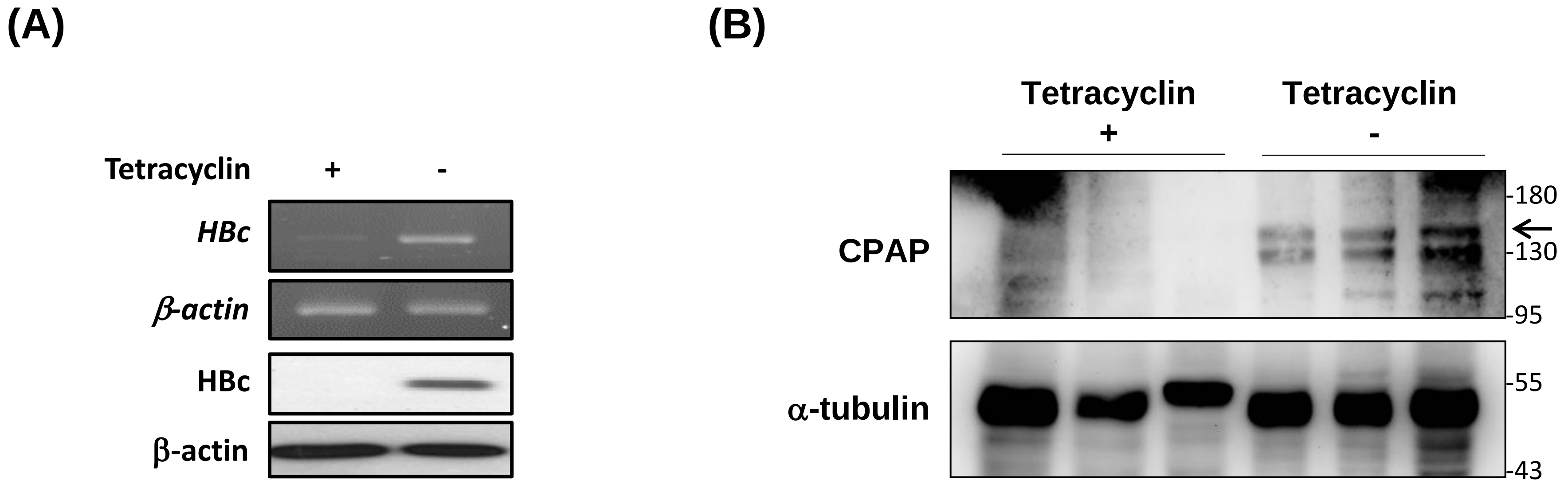

(A)
Tetracyclin
+
-
HBc
β-actin
HBc
β-actin
(B)
Tetracyclin
+
Tetracyclin
-
CPAP
α-tubulin
180
130
95
55
43

Figure S3

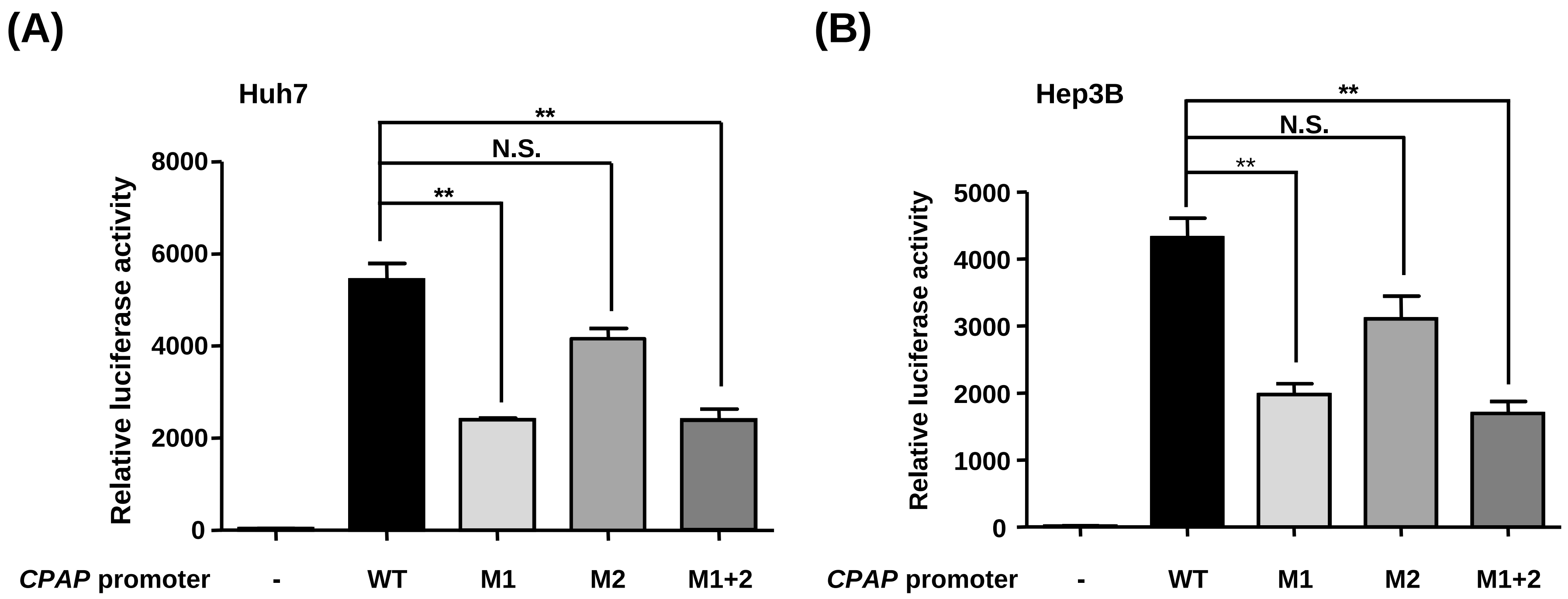
(A)
Huh7
Relative luciferase activity
8000
6000
4000
2000
0
**
N.S.
**
CPAP promoter
-
WT
M1
M2
M1+2
(B)
Hep3B
Relative luciferase activity
5000
4000
3000
2000
1000
0
**
N.S.
**
CPAP promoter
-
WT
M1
M2
M1+2

Figure S4

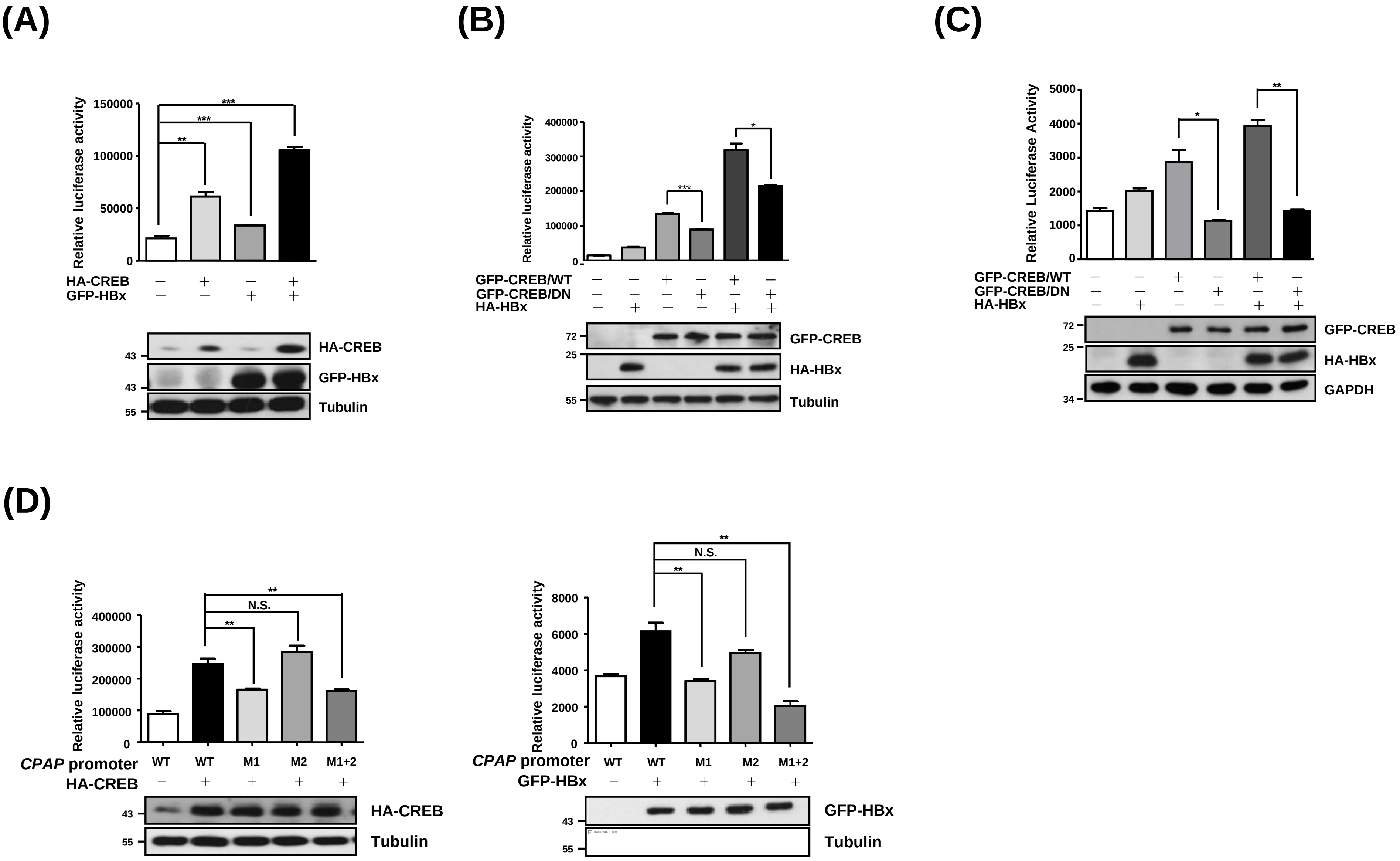
(A)
(B)
(C)
(D)
Relative luciferase activity
150000
100000
50000
0
***
***
**
HA-CREB
GFP-HBx
HA-CREB
GFP-HBx
Tubulin
43
43
55
400000
300000
200000
100000
0
*
***
GFP-CREB/WT
GFP-CREB/DN
HA-HBx
72
25
55
GFP-CREB
HA-HBx
Tubulin
Relative Luciferase Activity
5000
4000
3000
2000
1000
0
**
*
GFP-CREB/WT
GFP-CREB/DN
HA-HBx
72
25
34
GFP-CREB
HA-HBx
GAPDH
Relative luciferase activity
400000
300000
200000
100000
0
**
N.S.
**
CPAP promoter
WT
WT
M1
M2
M1+2
HA-CREB
43
55
HA-CREB
Tubulin
Relative luciferase activity
8000
6000
4000
2000
0
**
N.S.
**
CPAP promoter
WT
WT
M1
M2
M1+2
GFP-HBx
43
55
GFP-HBx
Tubulin

Figure S5

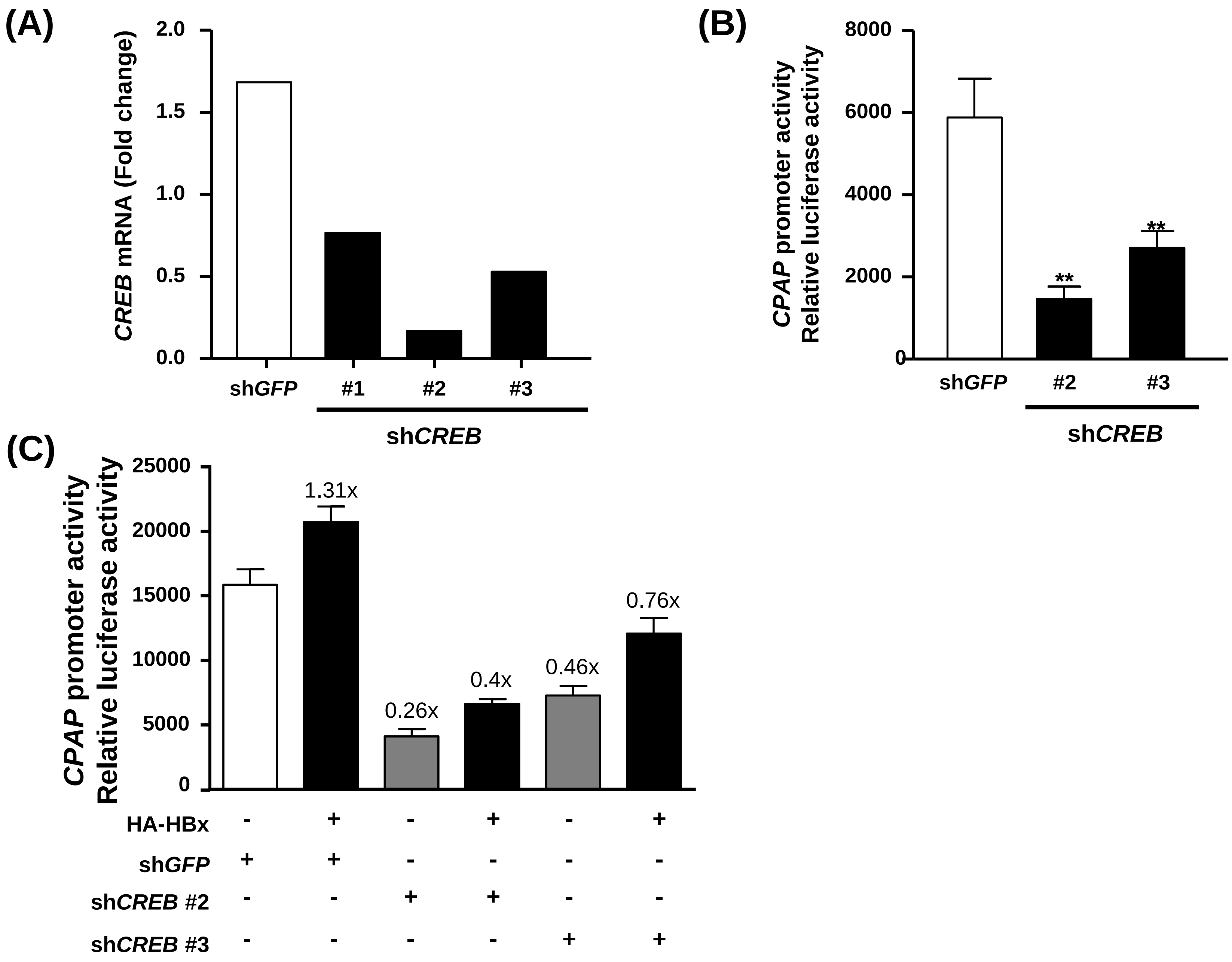

(A)
CREB mRNA (Fold change)
2.0
1.5
1.0
0.5
0.0
shGFP
#1
#2
#3
shCREB
(B)
CPAP promoter activity
Relative luciferase activity
8000
6000
4000
2000
0
**
**
shGFP
#2
#3
shCREB
(C)
CPAP promoter activity
Relative luciferase activity
25000
20000
15000
10000
5000
0
1.31x
0.26x
0.4x
0.46x
0.76x
HA-HBx - + - + - +
shGFP + + - - - -
shCREB #2 - - + + - -
shCREB #3 - - - - + +

Figure S6

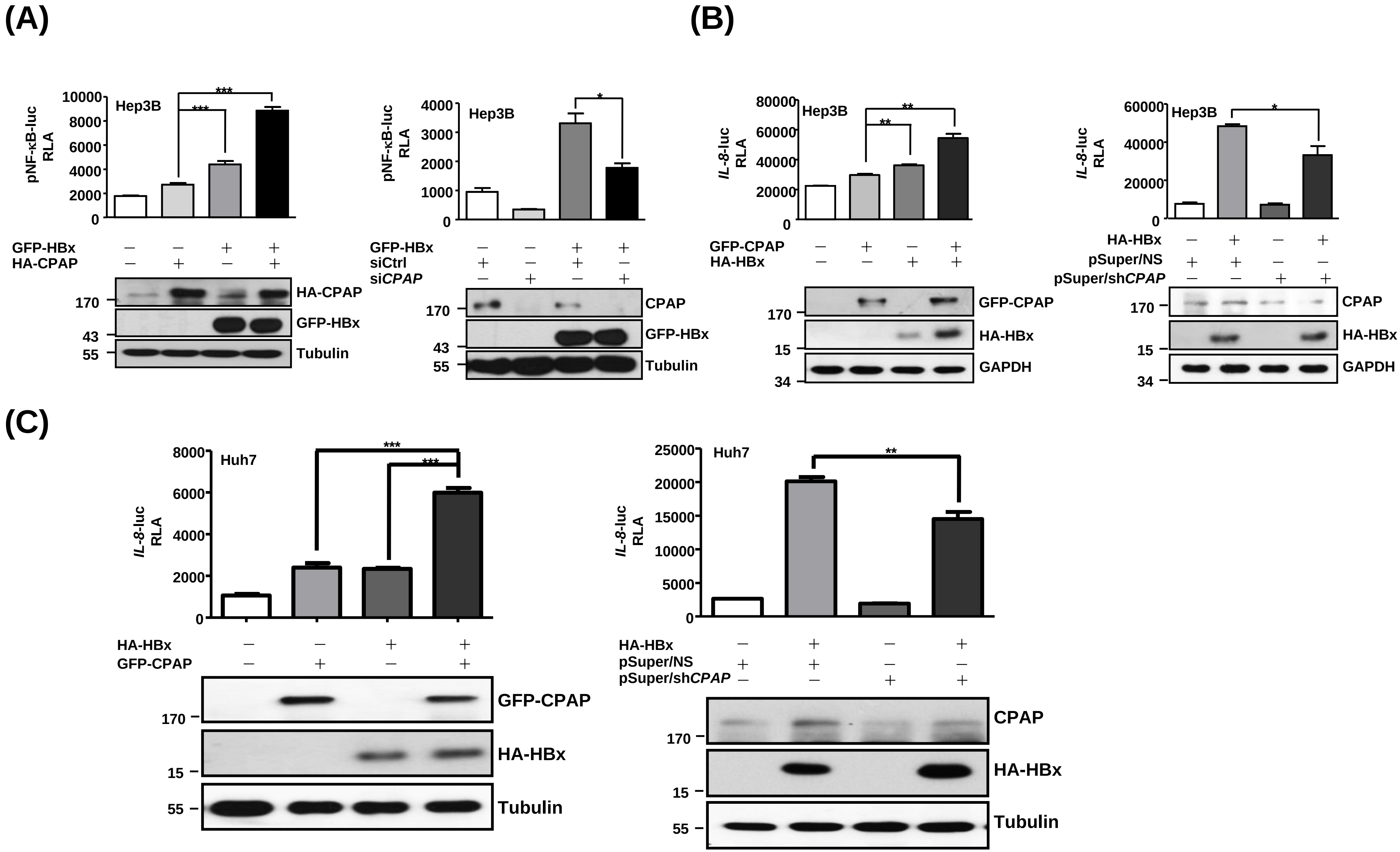

(A)
(B)
(C)
Hep3B
pNF-κB-luc RLA
GFP-HBx
HA-CPAP
siCtrl
siCPAP
HA-CPAP
GFP-HBx
Tubulin
CPAP
IL-8-luc RLA
GFP-CPAP
HA-HBx
pSuper/NS
pSuper/shCPAP
GAPDH
Huh7

Figure S7

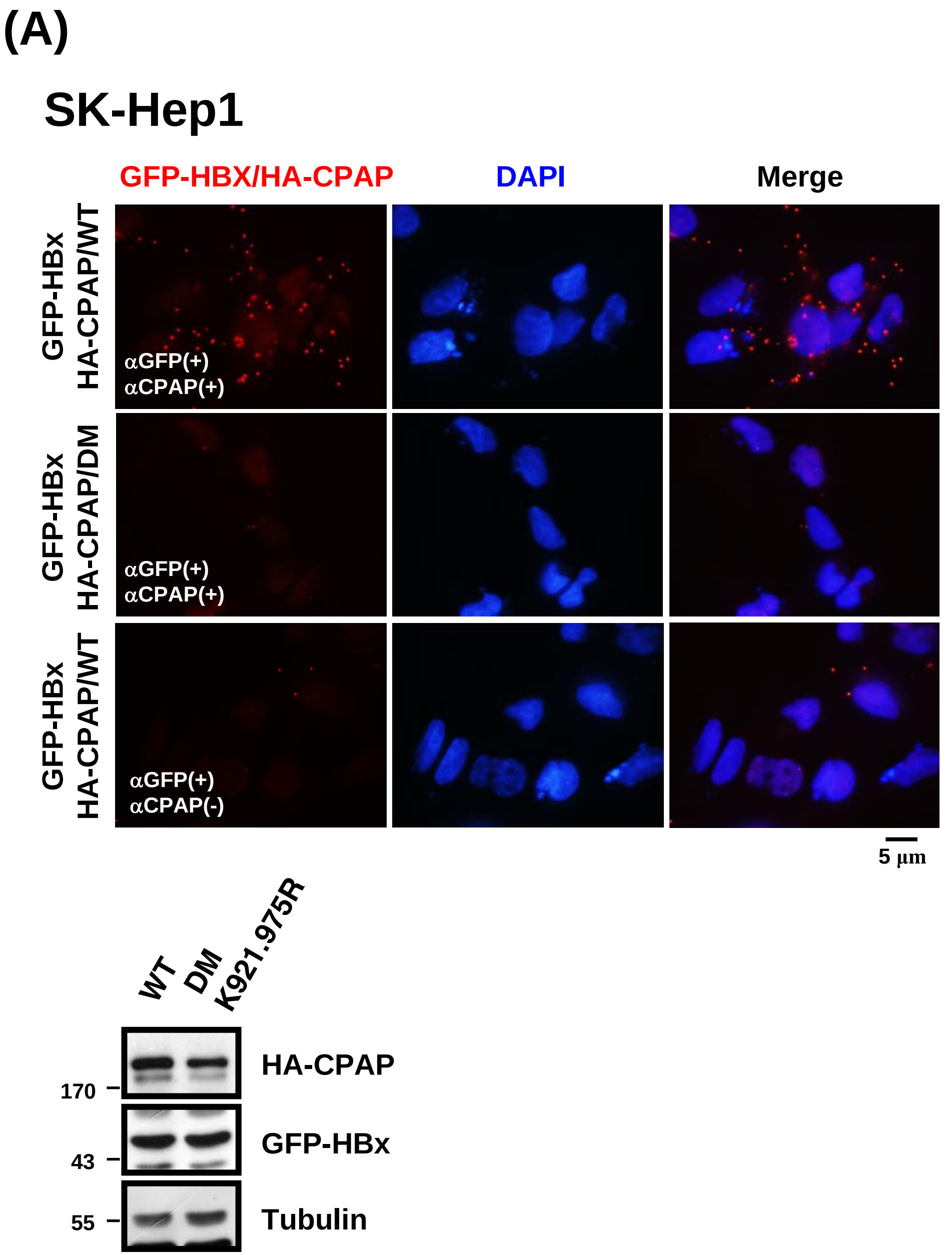
(A)
SK-Hep1
GFP-HBX/HA-CPAP
DAPI
Merge
GFP-HBx
HA-CPAP/WT
αGFP(+)
αCPAP(+)
GFP-HBx
HA-CPAP/DM
αGFP(+)
αCPAP(+)
GFP-HBx
HA-CPAP/WT
αGFP(+)
αCPAP(-)
5 μm
WT
DM
K921.975R
170
HA-CPAP
43
GFP-HBx
55
Tubulin

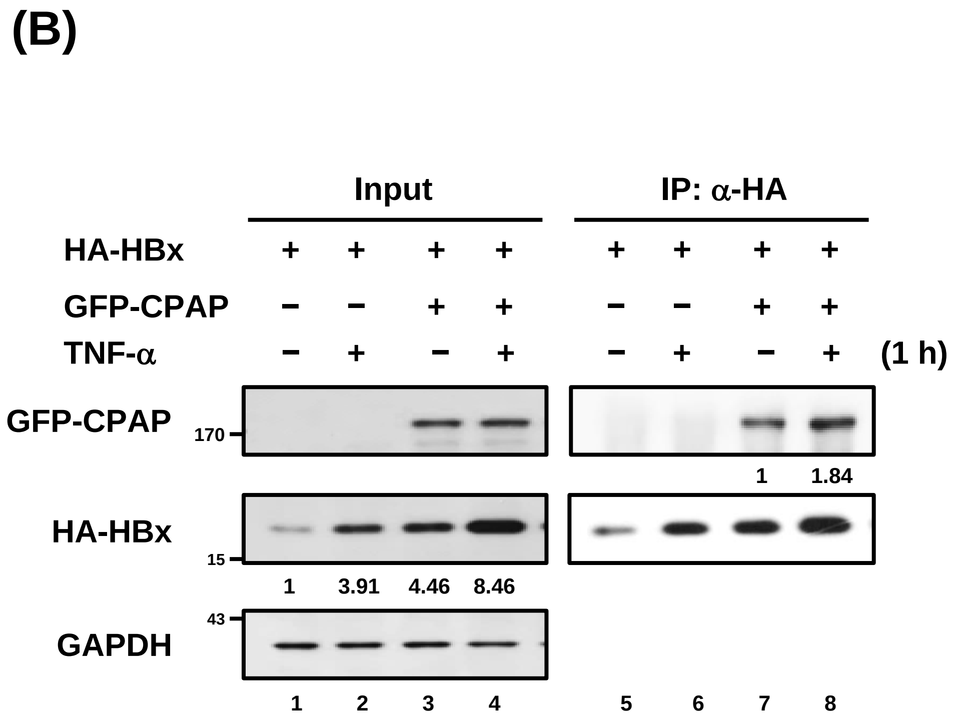
(B)
Input
IP: α-HA
HA-HBx
GFP-CPAP
TNF-α
(1 h)
GFP-CPAP
170
1
1.84
HA-HBx
15
1
3.91
4.46
8.46
43
GAPDH
1
2
3
4
5
6
7
8

Figure S8

(A)

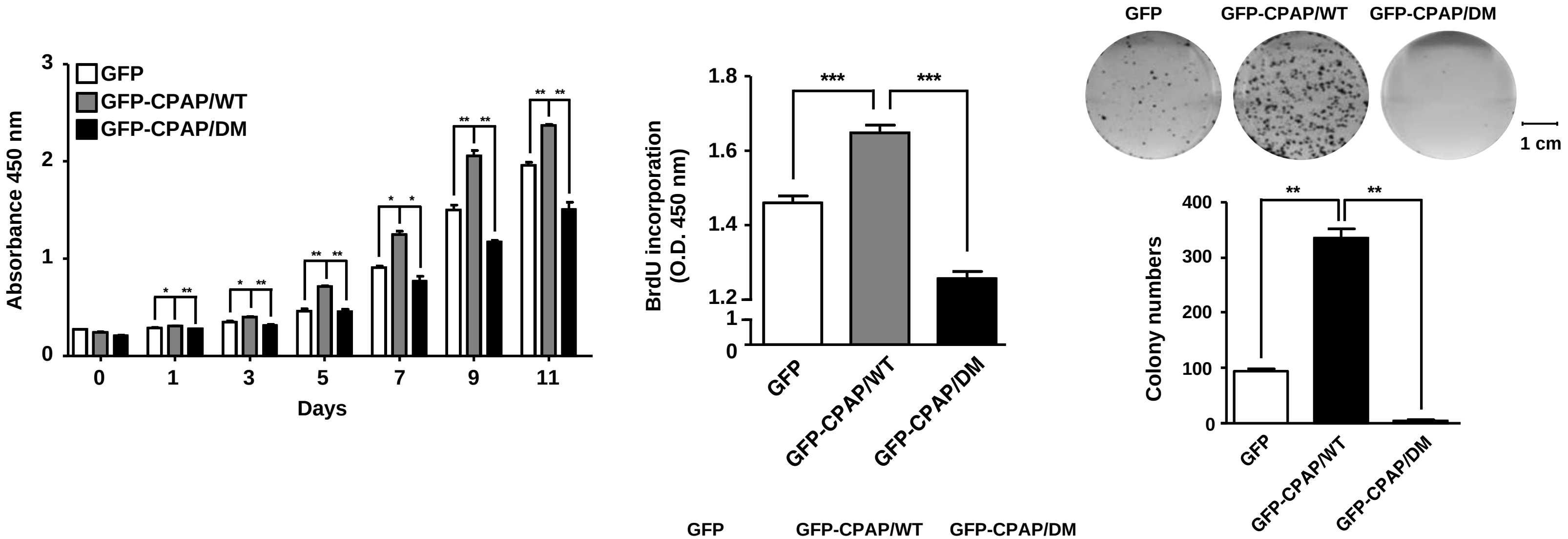
GFP
GFP-CPAP/WT
GFP-CPAP/DM
Absorbance 450 nm
Days
0
1
3
5
7
9
11
BrdU incorporation (O.D. 450 nm)
1.8
1.6
1.4
1.2
***
1 cm
Colony numbers
400
300
200
100
**

(B)

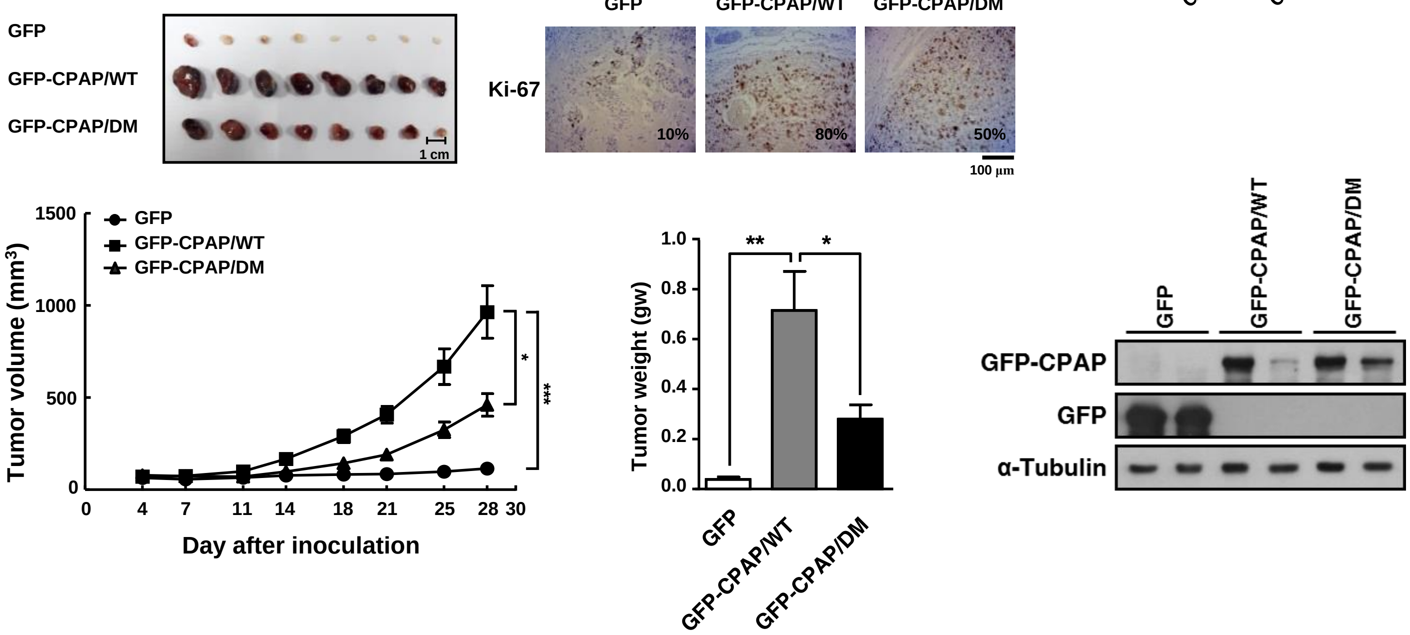
GFP
GFP-CPAP/WT
GFP-CPAP/DM
1 cm
Ki-67
10%
80%
50%
100 μm
Tumor volume (mm³)
1500
1000
500
Day after inoculation
4
7
11
14
18
21
25
28
30
Tumor weight (gw)
1.0
0.8
0.6
0.4
0.2
0.0
GFP-CPAP
α-Tubulin

Figure S9

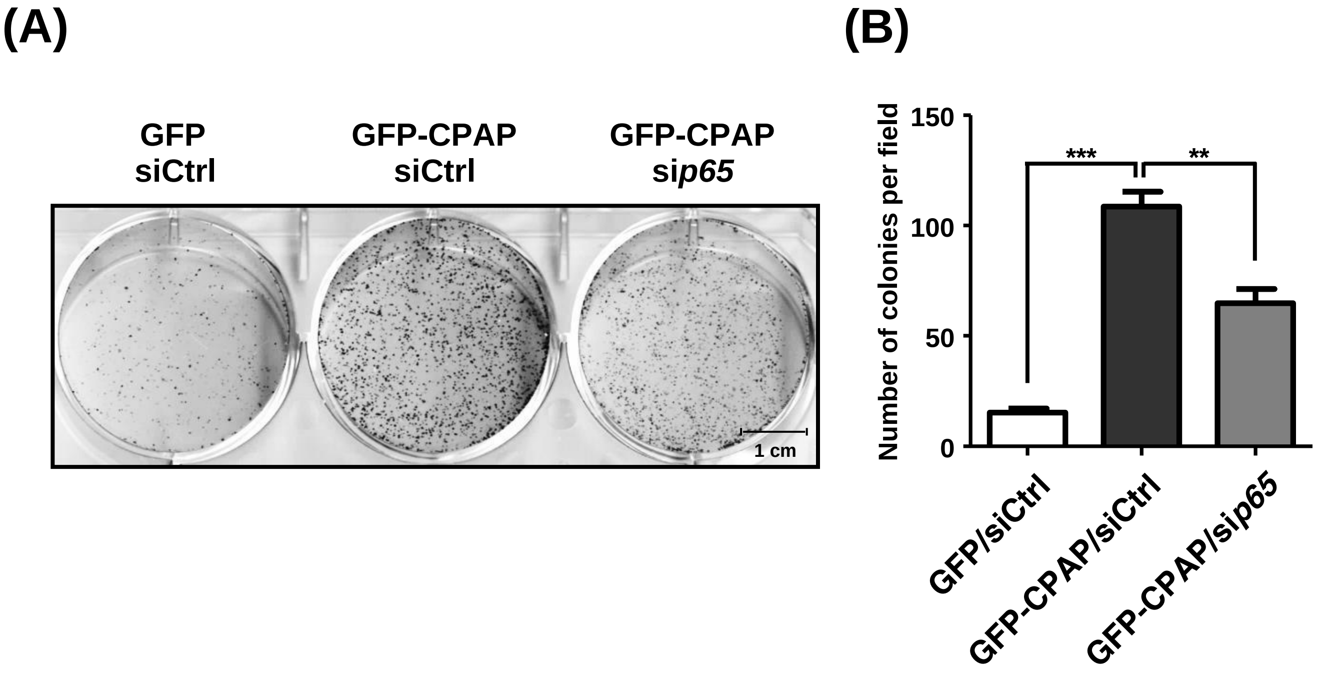
(A)
GFP
siCtrl
GFP-CPAP
siCtrl
GFP-CPAP
si*p65*
1 cm
(B)
Number of colonies per field
150
100
50
0
***
**
GFP/siCtrl
GFP-CPAP/siCtrl
GFP-CPAP/si*p65*

Figure S10

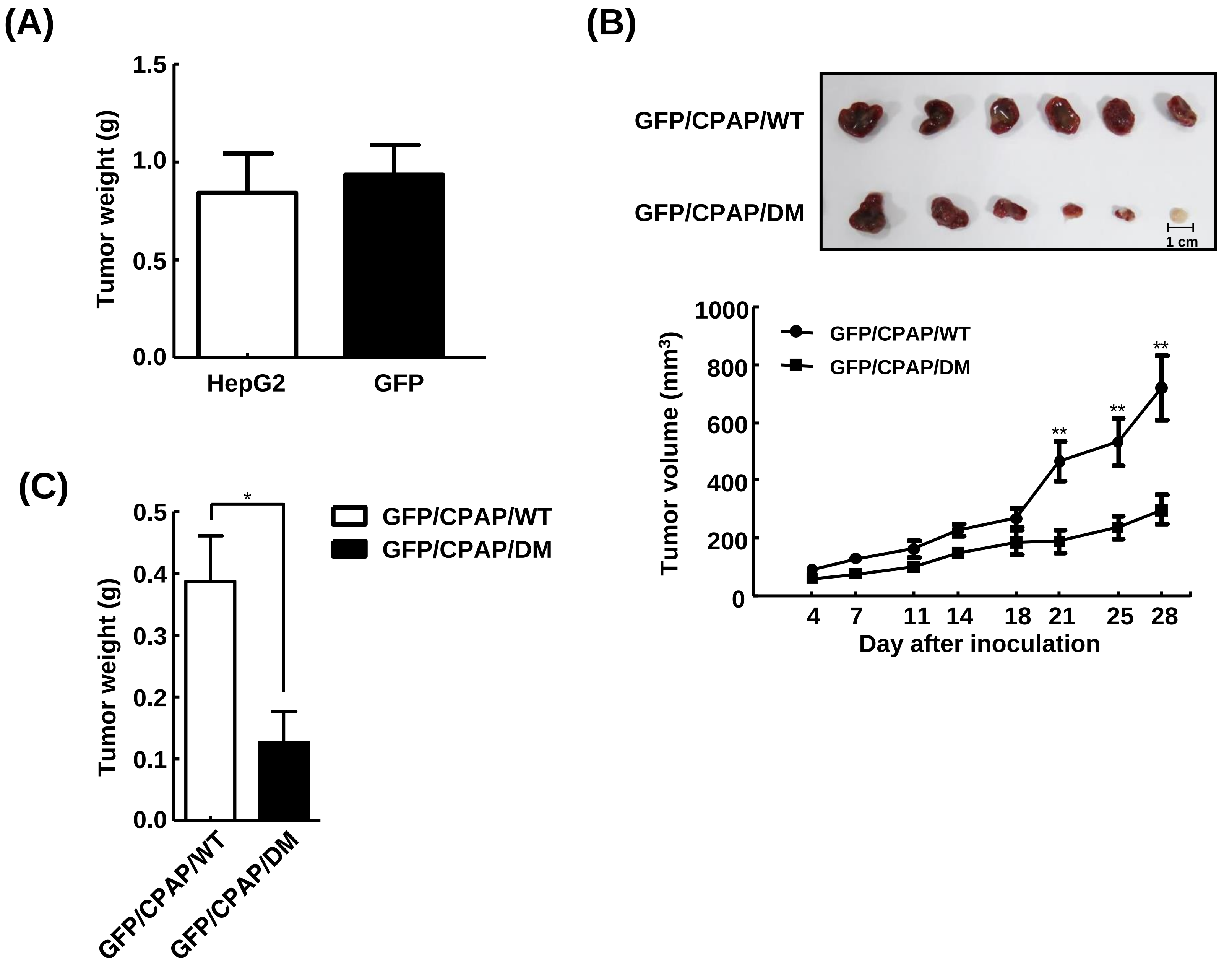
(A)
1.5
1.0
0.5
0.0
Tumor weight (g)
HepG2
GFP
(B)
GFP/CPAP/WT
GFP/CPAP/DM
1 cm
1000
800
600
400
200
0
Tumor volume (mm3)
GFP/CPAP/WT
GFP/CPAP/DM
**
**
**
4
7
11
14
18
21
25
28
Day after inoculation
(C)
0.5
0.4
0.3
0.2
0.1
0.0
Tumor weight (g)
*
GFP/CPAP/WT
GFP/CPAP/DM
GFP/CPAP/WT
GFP/CPAP/DM

Figure S11

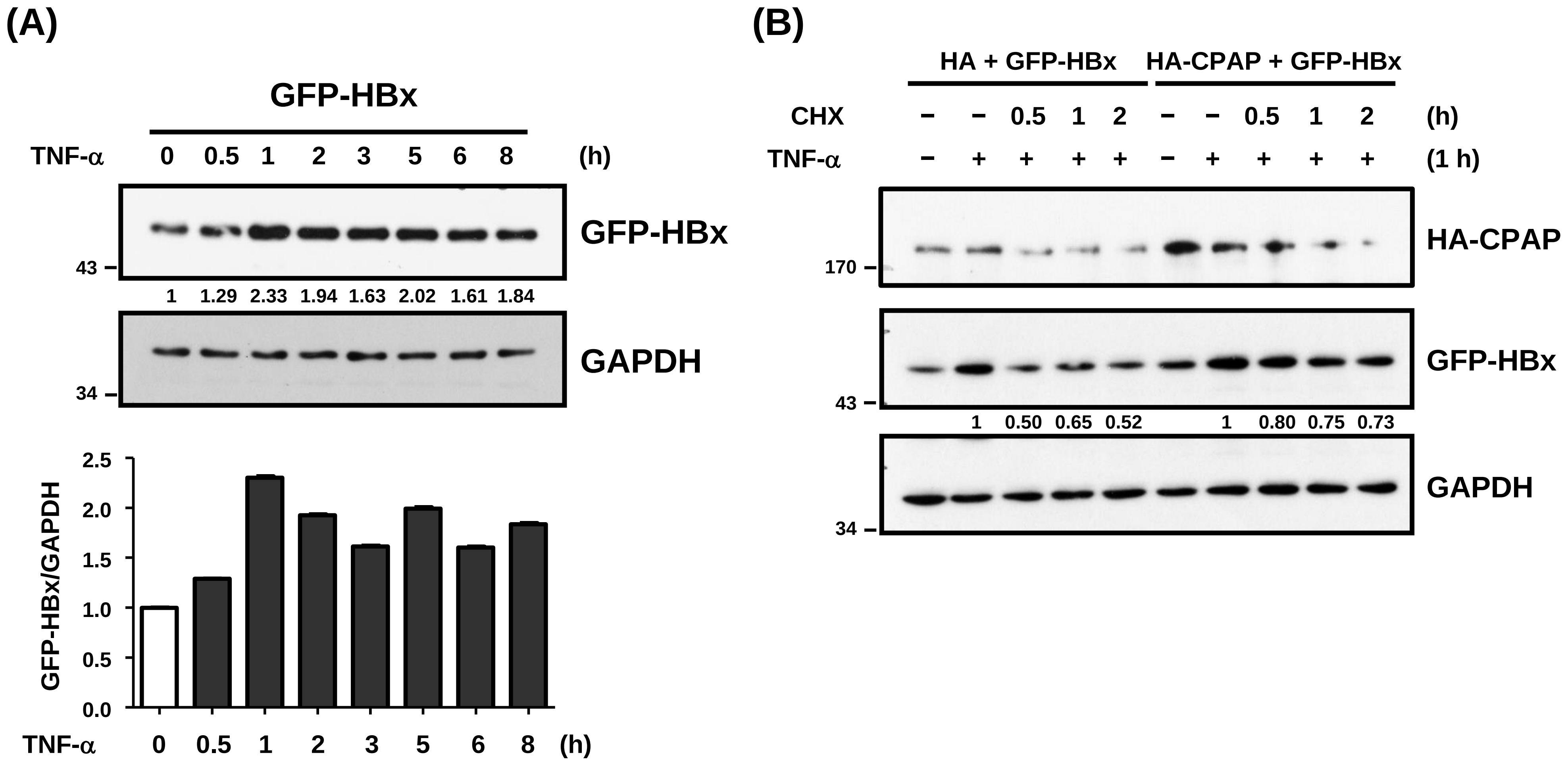
(A)
GFP-HBx
TNF-α 0 0.5 1 2 3 5 6 8 (h)
GFP-HBx
43
1 1.29 2.33 1.94 1.63 2.02 1.61 1.84
GAPDH
34
GFP-HBx/GAPDH
2.5
2.0
1.5
1.0
0.5
0.0
TNF-α 0 0.5 1 2 3 5 6 8 (h)
(B)
HA + GFP-HBx
HA-CPAP + GFP-HBx
CHX − − 0.5 1 2 − − 0.5 1 2 (h)
TNF-α − + + + + − + + + + (1 h)
HA-CPAP
170
GFP-HBx
43
1 0.50 0.65 0.52 1 0.80 0.75 0.73
GAPDH
34

Figure S12

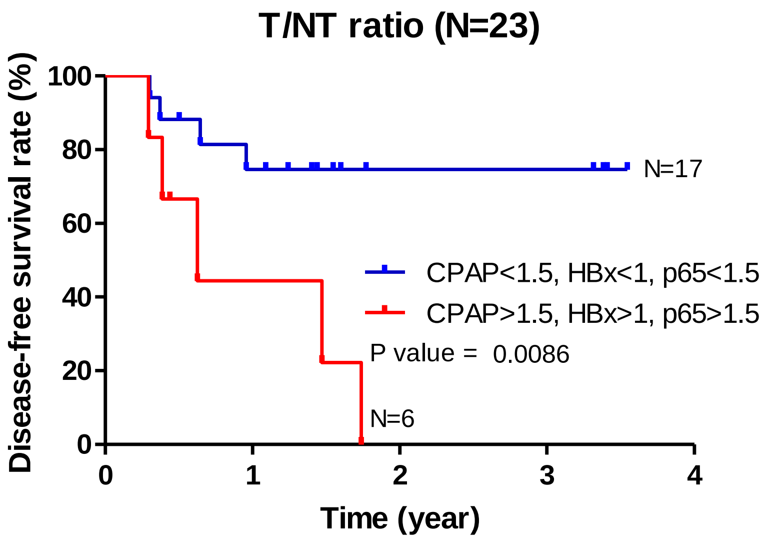

HR=5.006(1.325~18.911, p=0.018*)

Figure S13

(A)

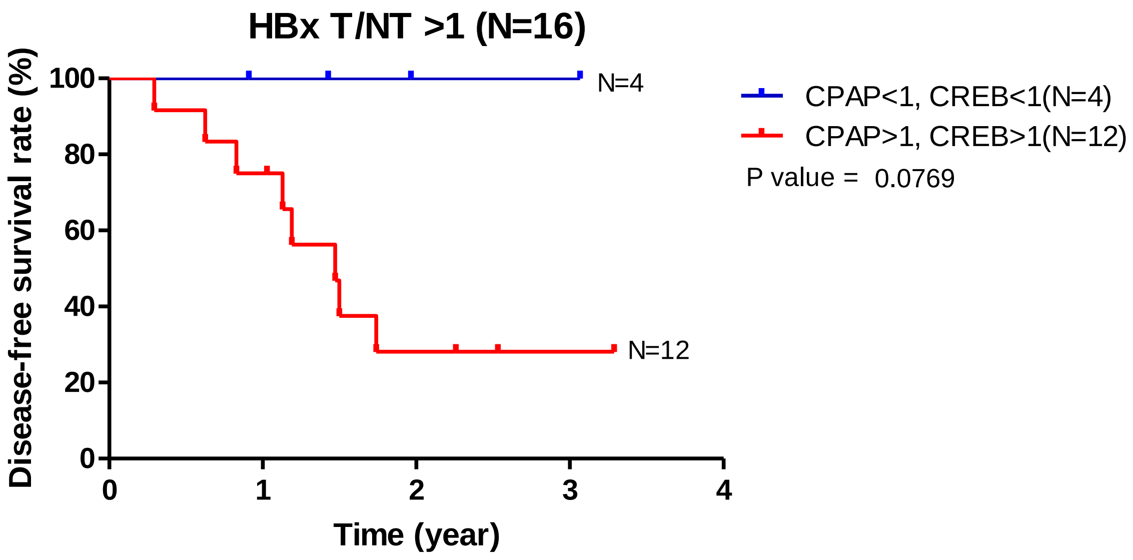

(B)

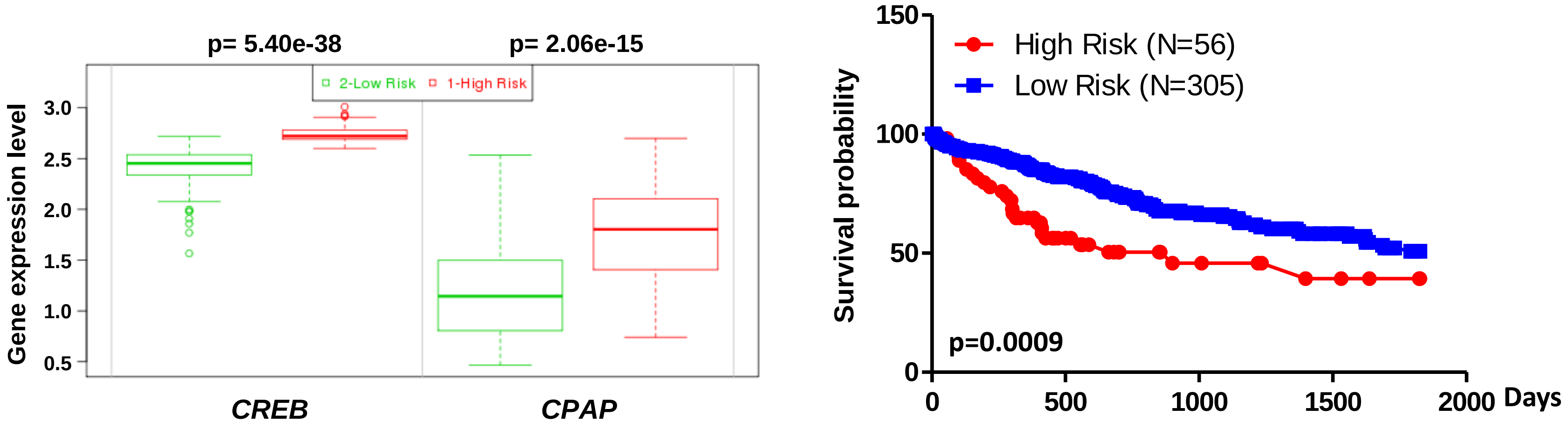

Supplement: Supplementary file 2 — Figure S1. HBx increases CPAP promoter activity in HCC cell lines. Figure S2. Expression of CPAP is increased in HBV genome-expressing HCC cells. Figure S3. The CREB binding site is crucial for CPAP promoter activity. Figure S4. CREB is essential for HBx-mediated CPAP promoter activity. Figure S5. Evaluation of CPAP promoter activity in shCREB transfected Hep3B cells. Figure S6. CPAP is involved in HBx-induced transcriptional activation of NF-κB. Figure S7. The interaction between CPAP and HBx is increased upon TNF-α treatment. Figure S8. CPAP promotes proliferation, colony formation, and tumorigenicity of HCC. Figure S9. NF-κB/p65 is essential for CPAP-mediated colony formation of HCC cells. Figure S10. Overexpression of CPAP/WT significantly increased tumor growth in a xenograft animal model. Figure S11. CPAP increases TNF-α-mediated HBx protein stabilization. Figure S12. The clinical outcome of overexpressed CPAP, HBx and activated NF-κB (p65) in HCC. Figure S13. Co-overexpression of CPAP and CREB is positively correlated with a poor disease-free survival rate in HBx-positive HCC. (PDF 1009 kb) [file 12929_2019_534_MOESM2_ESM.pdf]
